# The Developmental Influence of Primary Memory Capacity on Working Memory and Academic Achievement

**DOI:** 10.1037/a0039464

**Published:** 2015-06-15

**Authors:** Debbora Hall, Christopher Jarrold, John N. Towse, Amy L. Zarandi

**Affiliations:** 1 School of Experimental Psychology, University of Bristol; 2 Department of Psychology, Lancaster University; 3 School of Experimental Psychology, University of Bristol

**Keywords:** primary memory, working memory, mathematics, reading, managing distraction

## Abstract

In this study, we investigate the development of primary memory capacity among children. Children between the ages of 5 and 8 completed 3 novel tasks (split span, interleaved lists, and a modified free-recall task) that measured primary memory by estimating the number of items in the focus of attention that could be spontaneously recalled in serial order. These tasks were calibrated against traditional measures of simple and complex span. Clear age-related changes in these primary memory estimates were observed. There were marked individual differences in primary memory capacity, but each novel measure was predictive of simple span performance. Among older children, each measure shared variance with reading and mathematics performance, whereas for younger children, the interleaved lists task was the strongest single predictor of academic ability. We argue that these novel tasks have considerable potential for the measurement of primary memory capacity and provide new, complementary ways of measuring the transient memory processes that predict academic performance. The interleaved lists task also shared features with interference control tasks, and our findings suggest that young children have a particular difficulty in resisting distraction and that variance in the ability to resist distraction is also shared with measures of educational attainment.

Immediate memory is measured in terms of individuals’ ability to keep transient information active in memory, typically in correct serial order. In childhood, immediate memory performance is related to academic achievement (Bayliss, Jarrold, Gunn, & Baddeley, 2003; Bull, Espy & Wiebe, 2008). Contemporary research into immediate memory is often framed in terms of the related concept of working memory (Baddeley & Hitch, 1974; Conway, Jarrold, Kane, Miyake, & Towse, 2007), of which short-term storage is a core component (e.g., Alloway, Gathercole, & Pickering, 2006; Bayliss et al., 2003; Colom, Abad, Quiroga, Shih, & Flores-Mendoza, 2008; Engle, Tuholski, Laughlin, & Conway, 1999). Working memory tasks, in which participants maintain information (such as digit sequences) while completing a concurrent processing task, are thought to index retention processes in the face of distraction and are also linked to a wide variety of developing cognitive and academic skills (e.g., Bull et al., 2008; Yuill, Oakhill, & Parkin, 1989; Swanson & Alloway, 2012) and classroom behavior (Alloway, Gathercole, Kirkwood, & Elliott, 2009). Consequently, mapping the development of immediate memory performance in children in relation to working memory, and the causes of this development, is of considerable theoretical and practical importance.

In the present paper, we aim to carefully document the characteristics of immediate memory performance in children of different ages, and explore potential links between it and the earlier, but now increasingly influential, concept of primary memory. We use these conceptual links to motivate several new tasks and investigate their properties, using these data to help refine our theoretical understanding of immediate memory in children and its links to working memory and academic attainment.

## Immediate Memory and Academic Performance

Working memory tasks measure memory storage in the face of competing distraction and are thought to reflect a set of abilities, including immediate storage capacity, speed of processing, and executive control processes (Bayliss et al., 2003; Kane, Conway, Hambrick, & Engle, 2007; Unsworth, Redick, Heitz, Broadway, & Engle, 2009). Developmental improvements can be seen in general speed of processing (Kail & Ferrer, 2007) and executive control tasks (Alloway, Gathercole, Willis, & Adams, 2004). Furthermore, immediate storage capacity has been shown to increase between the ages of 4 and 15 years (Alloway et al., 2004). The growth observed in these domains has been linked to increases in working memory task performance during development, as well as increasing academic aptitude and general intelligence (Fry & Hale, 2000). Executive control in particular has received a great deal of attention, as working memory tasks are typically more predictive of academic performance than are measures of immediate storage or speed of processing (Bayliss et al., 2003; Swanson, 1994; Swanson & Alloway, 2012). However, attempts to fragment working memory tasks into their component parts have shown that processing speed is a predictor of classroom behavior (Jarrold, Mackett, & Hall, 2014), while immediate storage capacity predicts unique variance in reading ability (Bayliss et al., 2003) and mathematics (Bull et al., 2008). Indeed, Colom et al. (2008; see also Shahabi, Abad, & Colom, 2014) have suggested that immediate storage capacity alone underpins the link between working memory and academic attainment.

Reading is likely to tax the developing immediate memory system as multiple pieces of information must be managed online in order to decode words while building mental models of sentences for comprehension (Engel de Abreu & Gathercole, 2012; Wang & Gathercole, 2013). Similarly, mathematical problems require the concurrent storage of task instructions and running totals (Andersson, 2008; Kyttälä, Aunio, Lepola, & Hautamaki, 2014). Immediate memory capacity undoubtedly plays a role in successful use of working memory for this purpose, and valid measures of this construct must therefore be used to clarify the nature of any suggested relationship between academic performance and working memory.

Many studies examining this link have used immediate serial recall (ISR) span tasks to measure storage capacity in developing populations (e.g., Bayliss et al., 2003; Bull et al., 2008; Gathercole, Pickering, Knight & Stegmann, 2004); however, we argue that span tasks are not ideal measures of immediate memory, as they are inherently impure. It is probable that multiple systems underpin ISR performance, with competing theories implicating active portions of long-term memory, or the use of strategic skills and metamemory (cf. St. Clair-Thompson, 2007), all alongside any temporary memory storage system. Indeed, in work intended to isolate the “focus of attention” (Cowan, 2001; Oberauer, 2003) from any contribution of rehearsal or long-term memory when measuring immediate memory, Cowan, Elliott et al. (2005) observed that the number of items that could be held within the focus of attention was strongly related to aptitude measures. In the current work we therefore assess whether our novel measures, designed to specifically estimate and characterize immediate memory in the absence of the above confounding factors, are more predictive of academic ability than are standard ISR tasks in young children. ISR and novel tasks will be compared and contrasted, with the aim of determining whether better predictions of academic performance can be made if immediate memory can be isolated from strategic influences and long-term memory contributions.

In this study, we elected to focus solely on recall of verbal information. Immediate recall in the verbal domain has been heavily studied in relation to academic achievement in children (Bayliss et al., 2003; Bull et al., 2008; Gathercole, Alloway, Willis, & Adams, 2006), and may, potentially, rely on separate systems to those involved in visual or spatial immediate recall (Alloway, Gathercole, & Pickering, 2006; Smith & Jonides, 1997). Indeed, verbal and visuospatial immediate recall measures are dissociable from one another throughout development from age 4 to 15 (Alloway et al., 2004). Perhaps as a result of this, the associations between visual immediate memory and academic measures differ somewhat from those seen with verbal immediate recall (Holmes & Adams, 2006; Titz & Karbach, 2014). However, we note that while there is evidence for the separability of these two immediate memory systems, there is also support for the view that they share common processes and features (Chuah & Maybery, 1999; Cortis, Dent, Kennett, & Ward, 2014; Jones, Farrand, Stuart, & Morris, 1995), and it was these processes that we sought to capture in this work.

## Primary Memory

The terms *short-term memory* (STM) and *long-term memory* bring with them assumptions about the temporal properties of the memory system. However, this focus on duration (short and long) carries with it some ambiguity about the processes involved in retaining memory material. The current work therefore uses in preference Waugh and Norman’s (1965, see also James, 1890) definition of primary memory to characterize the “pure” capacity of immediate memory in the absence of additional contributions from rehearsal processes or long-term memory. In contrast to STM, primary memory carries with it theoretical assumptions about the processes involved in, and phenomena associated with, immediate memory recall. It therefore provides a potentially more informative framework in which to study and characterize immediate memory recall. The characteristics of primary memory include the fact that it accommodates the concurrent maintenance of a fixed number of items (Waugh & Norman, 1965; Unsworth & Engle, 2007), and that it is open to conscious awareness (James, 1890). Primary memory therefore maintains the subset of items that fall within some form of focus of “current attention” (Broadbent, 1958). However, recall from primary memory is also characterized by spontaneous serial ordering of the output, even when accurate serial order is not required (Broadbent, 1958; Bryden, 1971; Sperling, 1967). Secondary memory (which may be seen as a complement to long-term memory) is, on the other hand, characterized by recall that is not spontaneously serial ordered and is likely to be the product of a more controlled or probed search of items not within the current focus of attention. Shelton, Elliott, Matthews, Hill, and Gouvier (2010) have used structural equation modeling to show that primary memory, secondary memory, and working memory are indeed separable latent factors, which contribute differently to variation in fluid intelligence (see also Unsworth & Engle, 2007; Unsworth, Brewer, & Spillers, 2010).

This conceptualization of primary memory, therefore, obviously shares many similarities with more recent notions of the focus of attention in models of working memory (e.g., Cowan, Elliott et al., 2005; Cowan, Nugent, Elliott, Ponomarev, & Saults, 1999; Oberauer, 2003), which is the items held active in immediate memory, independent of sensory information (Cowan, 2011). However, we prefer the term primary memory because it has clearly defined processes and characteristics, that include the additional claim that recall from this system is characterized by spontaneous and accurate serial order output (Broadbent, 1958; Bryden, 1971; Sperling, 1967).

There are also two reasons why a focus on primary memory is timely and conceptually significant (see also, Unsworth et al., 2007). First, few studies have directly addressed the development of primary memory, with those that have done so recently producing conflicting results (De Alwis, Myerson, Hershey, & Hale, 2009; Jarrold et al., 2013; Roome, Towse, & Jarrold, 2014). Second, we chose to focus on primary memory to adapt existing (adult-based) paradigms that capture significant elements of this concept. We modified a dichotic listening paradigm, which was originally used by Broadbent (1958) to reveal the differences between primary memory and perceptual attention. Following Waugh and Norman (1965), immediate and probed free-recall tasks were also used to provide potential indices of primary memory capacity. As such, the novel tasks in this paper have been developed to specifically index spontaneous serial ordering of material that is within the focus of current attention.

### Measuring Primary Memory Capacity by Adapting Dichotic Listening Paradigms

In a typical dichotic listening experiment, participants are presented with items to both ears simultaneously but are required to attend to only one stream. Bryden (1971) showed that when adult participants were required to freely recall from the attended ear (with four items presented to each ear), recall was equally good at all serial positions, with high probability of spontaneous serial order output. However, when participants were required to recall from the unattended ear (whether before or after the attended items), recall followed a steep recency curve, with a clear advantage for the final item. This suggests that the attended items were held within primary memory, and that the unattended items were held within a separate store, such as perceptual memory (cf. Bhatarah, Ward, & Tan, 2006; Broadbent, 1958; Lachter, Forster, & Ruthruff, 2004). Indeed, findings from earlier, similar, dichotic listening studies formed the basis of Broadbent’s (1958; see also Sperling, 1967) theory of perception and memory, as the characteristics of recall from different streams evidenced separate stores (in his terms, S-system [sensory] and the P-system [limited capacity channel]). A focus on the processes involved in primary memory, therefore, provides a means of determining which items are held within primary memory and which are held within a separate system. For example, there are individual differences in performance on dichotic listening tasks, with adult participants who have low to average digit spans showing the pattern observed by Bryden (1971), while participants with higher spans show equally good recall across items heard in either ear (Parkinson, 1974). This suggests that successful recall from an unattended stream is possible when the number of attended items does not exhaust primary memory capacity (see also Colflesh & Conway, 2007, for links between working memory capacity and unshadowed speech perception).

We therefore developed a new selective free-recall memory task, drawing on the logic of these dichotic listening studies, to provide a potential index of primary memory in our sample. This involved sequentially presented verbal items, rather than simultaneous presentation of items to each ear, in order to make the task suitable for use with young children. Specifically, two memory lists were interleaved with one another, with alternate items being “focal” and “nonfocal.” Children were told to remember only the focal items; hence, the task will be referred to as the *interleaved lists* task. This procedure was piloted in a group of 6-year-old children prior to use in the current experiment and produced patterns of data that were analogous to those found using dichotic listening presentation in adults; specifically, focal items were more likely to be recalled, and were more likely to be spontaneously recalled in accurate serial order, than were nonfocal items, even though children were not told to serially order their output (these pilot data can be found in supplementary materials; see also Roome et al., 2014, for data on this task in the visual modality, and Cowan, AuBuchon, Gilchrist, Ricker, & Saults, 2011, for an analogous visual working memory paradigm). It is worth noting that this task potentially requires the inhibition or removal of the interleaved nonfocal items, a point that we return to in the Discussion section. However, Cowan and colleagues (e.g., Cowan, Elliott et al., 2005; Cowan, AuBuchon et al., 2011) have shown that methods requiring selective attention to a single stream do index a consistent number of items in the current focus of attention. Those previous data, our own pilot data, and the body of work on dichotic listening tasks reviewed earlier therefore suggest that it is appropriate to explore this kind of task as a potential index of primary memory.

## Measuring Primary Memory Capacity Using Probed Free Recall

Waugh and Norman (1965) and others (e.g., Murdock, 1968) have used recall of the final list items in immediate free recall to estimate primary memory capacity. However, a potential problem with this approach is that it relies on participants beginning their recall with these list-final items, which is not always the case (see Howard & Kahana, 1999). One way of enforcing this response pattern is by setting memory probes at various points from the end of the list. For example, Waugh and Norman (1965) gave participants a probe digit to request recall of the last two, three, four, or five items of a 10-item list and examined the difference in recall of these probed items versus items from the earlier section of the list. Raymond (1969; see also Aslan, Bäuml, & Grundgeiger, 2007; Murdock, 1968; Waugh & Norman, 1965) suggested that once any one set of memory items has been probed from a section of a list, the resulting output interference from that recall set would result in all subsequently probed items from the same list being drawn from secondary memory. Therefore, there is a potential difference in the characteristics of items that are recalled from the last section of a list and those that are recalled from the first section (cf. Tulving & Colotla, 1970), particularly when the last section is probed first. In addition, if items from the last section of a list are maintained in primary memory, one would expect a high degree of spontaneous serial ordering of these items when the last section of the list is probed first. The difference in performance on items recalled from the last section when probed first, as opposed to when probed second, can therefore potentially index which items are drawn from primary memory.

This assumption formed the basis of our second new measure, the split span task, which was again piloted prior to use in the current experiment. The key findings from the pilot data were that items from the last section of the list, when probed first, were recalled more accurately and with a greater degree of spontaneous serial ordering than were either items from the last section when probed second, or items from the first section whether probed first or second. There was also some evidence of an increase in primary memory capacity in older as opposed to younger children, and a clear increase in the likelihood of older children starting their recall from the start of a probed list (these data can be found in the Supplementary Materials). These data suggested that it was appropriate to assume that items from the “last” portion of the list, when probed first, were drawn from primary memory.

## Estimating Primary Memory From Free Recall

An alternative method for estimating primary memory from immediate free recall follows from recent work by Ward, Tan, and Grenfell-Essam (2010). These authors tested adults on free recall of unpredictable list lengths of between one and 15 items. When participants recalled lists of one to five items, there was no large change in probability of item recall across serial positions. On longer lists, the serial position curve became clearly bowed, with increasingly steep recency portions of the curve from six-item lists onward. In addition, Ward et al. found that participants were likely to begin recall with the first item on the list until lists exceeded five items in length, at which point they were more likely to begin recall with items from the end of the list. One reading of these data (cf. Farrell, 2010), is that the capacity of primary memory can be referenced from immediate free-recall data both by the point at which the serial position curve becomes nonflat, and the point at which a participant stops recalling the first list item first as list length increases.^1^ Bowing and flatness of serial position curves are unlikely to be observed fully using ISR span methods, and using varying list lengths in a free-recall methodology allows us a greater insight into cognitive processes underpinning performance at short and long list lengths (see Gibson et al., 2013; Unsworth & Engle, 2006). In the current work, we therefore examined these characteristics using Ward et al.’s (2010) varying list length methodology with children.

## The Current Work

The present experiment was designed with three main objectives in mind, which combined methodological and theoretical issues: (a) to triangulate performance on our three novel measures of primary memory capacity to determine whether a consistent estimate of primary memory could be achieved in children, (b) to examine the developmental change in these measures so as to derive potential indices of primary memory from the same set of tasks in two age groups of children, and (c) to test whether these novel measures of primary memory capacity can better and/or separately predict storage contributions to working memory tasks and academic achievement than traditional ISR-based span measures of STM.

Following the previous discussion, we assumed that the defining characteristics of primary memory are (a) flat, comparable performance across items in the serial position curve (cf. Unsworth & Engle, 2006); and (b) evidence that participants begin recall (probability of first recall) with the first item on the list, which we take as a marker of an intention to recall in forward serial order (Broadbent, 1958; Farrell, 2012; Sperling, 1967; Ward et al., 2010). Given this, and to properly examine the extent to which participants spontaneously elected to recall the items in serial order, all three novel tasks were based on free-recall methodologies. In addition, list length was unknown prior to presentation for any trial. This manipulation was put in place to ensure that children could not selectively attend to list items, with a view to minimizing strategic contributions to primary memory estimation which may be a further potential issue with ISR span tasks (St. Clair-Thompson, 2007, though see Jarrold & Hall, 2013).

To that end, in this experiment modified versions of the previously piloted interleaved lists and split span tasks were used alongside a free-recall task with unpredictably varying list lengths, as well as traditional simple span and complex span tasks. Standardized assessments of reading and mathematics were also given to participants, who were a cross-sectional sample of children in the early primary school years. Specifically, groups of Year 1 (aged 5 to 6) and Year 3 (aged 7 to 8) children were tested. Evidence for a developmental change in primary memory capacity was expected to be observed in each of the new memory measures in this study, as indexed by the average number of items recalled in a task and the probability of recalling the first item first (as an index of spontaneous serial ordering).

## Method

### Participants

A power analysis was conducted on a relevant existing data set, namely that provided by Tam, Jarrold, Baddeley, and Sabatos-DeVito (2010). These authors examined STM in children in the same two year groups as the current study, specifically Year 1 (*M*_age_ [117] = 12.78, *SD* = 2.61) and Year 3 (*M*_age_ [114] = 16.23, *SD* = 2.90). They found significant age related changes in ISR digit span performance, and an analysis using G*Power (Faul, Erdfelder, Lang, & Buchner, 2007), revealed that a total sample of 34 would be required to detect large effects of this nature (Cohen’s *d* = .64), when using a one way ANOVA with 95% power and alpha set at .05. In order to exceed these numbers and to provide an appropriately large sample for correlational analyses we requested consent from all parents of children in Years 1 and 3 of four local primary schools. All of the 101 children for whom full parental consent was obtained were tested.

The resultant sample consisted of 50 Year 1 pupils (23 males, mean age 6 years 4 months, range 5 years 10 months to 6 years 10 months) and 51 Year 3 pupils (27 males, mean age 8 years 5 months, range 7 years 10 months to 8 years 11 months). All participants completed the experimental memory tasks, with the exception of one individual in Year 1 who was absent for the session in which the split span task was presented. However, further absences at the time when the reading and mathematics assessments were given meant that a full data set that also included these measures’ data was only available for 92 children (43 in Year 1, 49 in Year 3). As a result, in the analyses presented below performance on the experimental tasks is examined in the full data set, but the correlational analyses examining the relationships between these measures and academic attainment is conducted on the subset of 92 participants who provided data on every task.

As a note on data exclusions and methodology used in this study, we would like to state clearly that “We report how we determined our sample size, all data exclusions (if any), all manipulations, and all measures in the study” (Simmons, Nelson, & Simonsohn, 2012).

### Design

Each child completed three individual testing sessions lasting approximately 30 min each. In each of the first two sessions, children completed two memory tasks, and in the final session, they were tested on one memory task; these tasks were presented to all children in the order in which they are introduced next. In addition to the memory measures, all children were tested on the Sentence Completion Forms of the NFER-Nelson (1998) Group Reading Test II Form A (6–14) and the age-appropriate test from the NFER-Nelson (1994) Mathematics 6–14 series in separate sessions. The Group Reading Test spans a wide age range and was administered to all children in both year groups. The Mathematics 6–14 test uses different test questions dependent on children’s level of education, and the appropriate tests were given to each age group (Progress in Maths 6 was given to Year 1, and Progress in Maths 8 was given to Year 3 children). Both reading and mathematics assessments give a fairly broad overview of skill in each area. The reading assessment indexes word reading and sentence comprehension, and the same questions are given to both age groups. The mathematics assessment taps proficiency in facts and procedures, concepts, problem solving, and reasoning, with identical subscales for both age groups.

### Tasks and Procedure

All memory tasks were programmed using Runtime Revolution software and presented on Macintosh Powerbook and MacBook computers. A total of 348 words were used in the memory tasks, which were single syllable concrete nouns, with age of acquisition of under 6.2 years (statistics from the Medical Research Council database, Wilson, 1988). Each word was paired with a color cartoon image. No words were repeated within or between tasks in a single testing session. All audio material was presented through the internal laptop speakers using male voices.

#### Simple span

Children were presented with increasing lists of words (two to eight) with five trials at each list length. If children successfully remembered any one list in correct serial order from the five trials at a given list length, they moved on to the next list length. If they failed to recall all lists within a given list length, testing was terminated at that point. The predictable list lengths and continuation rules used in this study enabled a direct comparison of the span tasks against existing literature on the link between working memory and academic achievement (see, e.g., Bayliss et al., 2003). Children were presented with a digital audio recording of words in a male voice and 3-cm-high color illustrations of those words for 1,000 ms in the center of the computer screen. A blank screen appeared briefly between each word. At the end of each trial, a cartoon giraffe appeared alongside a question mark and children were prompted to recall the words in the order they had heard them.

#### Complex span

This task followed the span procedure used in the simple span task (with the same number of trials at each of the same list lengths, and the same continuation and stopping criteria), but using digits. Digits were presented in a male voice as digital audio recordings, simultaneously with the appearance of the item in black in the center of the computer screen measuring approximately 2 cm high for 1,000 ms. Between each digit, children were presented with a large colored circle measuring around 4 cm high (either brown, pink, or blue) in the center of the computer screen and told to name the color of the circle as quickly as they could, similar to a complex span task used by Camos and Barrouillet (2011). As soon as the participant had named the circle, the experimenter tapped the spacebar of the computer, and the computer moved on to another colored circle. This processing task automatically ended after 3,000 ms, regardless of the number of circles that the child had named in that time. This task was therefore designed to fill the fixed 3-s processing window with near-continuous verbal distraction. The child was then presented with the next digit in the list. A cartoon dinosaur with a question mark over his head appeared when the participant was required to begin recall, and they were told to recall the digits in the order they had heard them.

#### Free-recall task

Participants were presented with word lists spoken in a male voice ranging from two to eight items in length, with five trials at each list length, giving a total of 35 trials. In contrast to the two span tasks just described, list lengths were pseudorandomly organized in five testing blocks, so that list length was unknown to the child before presentation of any given list. Children were presented with a cartoon penguin and told that he had words for them to remember. The word lists were then presented with the penguin in the corner of the screen and a speech bubble coming from his mouth in the center of the screen. An audio recording of each word simultaneous with a 3-cm-high color illustration of the word was presented in the center of the speech bubble for 1,000 ms, followed briefly by a blank screen and the next word. At the end of each trial, a question mark appeared above the penguin’s head, and children were asked to recall as many words as they could, in any order.

#### Interleaved lists

Children were introduced to two cartoon characters, SpongeBob and Patrick, who were identified by illustrations and two distinct male voices. They were explicitly told to pay attention to SpongeBob (i.e., focal stimuli) and try to remember his words in any order, and to try to ignore Patrick (nonfocal items). Four conditions corresponding to total list lengths three, four, five, and six were presented. Focal items were always presented first in sequence, and focal and nonfocal items were interleaved with one another, for example, the presentation order for a three-item list was focal—nonfocal—focal, with two focal items and one nonfocal item (four-item lists had two focal and two nonfocal items, five-item lists had three focal and two nonfocal items, and six-item lists had three focal and three nonfocal items). Items in each sequence were presented pictorially with a color cartoon image of the word, accompanied by an audio recording of one of two different male voices (one for SpongeBob and one for Patrick). SpongeBob always appeared on the left bottom corner of the screen and Patrick on the right. Images were presented in the center of the screen in a speech bubble originating from the relevant character’s mouth, and were displayed for 1,000 ms, with a 250-ms pause between each word. Children were only asked to recall focal items in order to maximize the chances of participants maintaining just the focal items in primary memory. There were five trials in each condition and these were all pseudorandomly organized within five blocks, so that children did not anticipate list length. After presentation of each trial, SpongeBob’s speech bubble reappeared in the center of the screen, highlighted in red alongside SpongeBob, which signaled that the child should try to recall the focal words only.

#### Split span

Children were presented with two cartoon characters, Charlie Cat and Danny Dog, which were identifiable by corresponding illustrations and two distinct male voices. In this task, six words were presented in each trial and the words were split between each character. The subset conditions were formed by the systematic manipulation of the two set lengths, giving Conditions 5:1, 4:2, 3:3, 2:4, and 1:5, where the first digit corresponds to the number of items in Set A presented by Charlie Cat and the second to the number of items in Set B presented by Danny Dog. Audio recordings of each word were presented simultaneously with 3-cm-high color illustrations in the center of the computer screen for 1,000 ms with the related character to the side of the screen (left for Charlie—Set A and right for Danny—Set B). After the word list had been presented, children were probed to recall either Charlie or Danny’s words first by presentation of the relevant character on the screen. After this first probe, children were then probed for the remaining character’s words with an image of that character on the screen. Children were told that they could recall words within a subset in any order. There were 10 trials in each subset condition, with five trials probing recall of Set A first and five trials probing recall of Set B first, which resulted in a total of 50 trials.

### Analysis

In the free-recall task, the interleaved lists, and the split span tasks, two key measures were extracted from the data. These were the average number of items recalled from the relevant memory set and the sum of the probability of items in serial position 1 being recalled first. For reasons that will be described below, the average number of items recalled from the free-recall task was derived from trials with at least five list items; the probability of first recall variable was extracted from all free-recall trials. In the interleaved lists task, recall of focal items was the key dependent variable, as this was found to be a potential indicator of primary memory capacity in our pilot work (see Supplementary Materials). In the split span task, the key dependent variable was the recall of Set B items when recalled first (the Supplementary Materials report evidence that first recall of this set was the best indicator of primary memory capacity). Partial credit scoring was used to calculate span in the simple span and complex span tasks (cf. Conway et al., 2005, who recommend this method for scoring span tasks as it is the most psychometrically appropriate of a range of options). Under this method, proportional credit is given for each item recalled at the correct serial position in any list. For example, in a list of three items, each item has a potential proportional score of .333. Summed proportional scores are then totaled across all trials within the span task. Total raw score was taken as the dependent variable on both the reading and mathematics assessments.

## Results

A summary of the descriptive statistics for all variables is presented in Table 1. Reliability estimates for the memory and academic measures are also shown, which were derived by computing Cronbach’s alpha.^2^ All reliability estimates were satisfactory to good, with most above .75. The results section is split into three parts. First, we examine the average number of items recalled and the probability of first recall of the first item on the just-presented memory list, on a task-by-task basis, to determine whether there were age differences in performance. Second, we compare estimates of primary memory across the whole sample to assess whether similar estimates of capacity were derived from the novel measures. Third, we consider individual differences in performance, exploring the predictive validity of the novel experimental tasks for simple and complex span performance, and for academic achievement.[Table-anchor tbl1]

### Task Analyses

#### Span tasks

Performance in the simple and complex span tasks was compared using a 2 × 2 mixed ANOVA, with year group as the between-subjects factor and task as a within-subjects factor. There was a significant main effect of task, *F*(1, 99) = 687.335, *p* < .001, *MSE* = 5.128, η_*p*_^*2*^ = .874, which reflected lower scores in complex span than simple span for both age groups. There was also a significant main effect of year group, *F*(1, 99) = 48.679, *p* < .001, *MSE* = 12.178, η_*p*_^*2*^ = .330, as Year 3 children achieved significantly higher span scores than Year 1 children across the two tasks. The interaction between task and year group was not significant, *F*(1, 99) = 1.097, *p* = .297, *MSE* = 5.128, η_*p*_^*2*^ = .011.

#### Free recall

**Figure 1 fig1:**
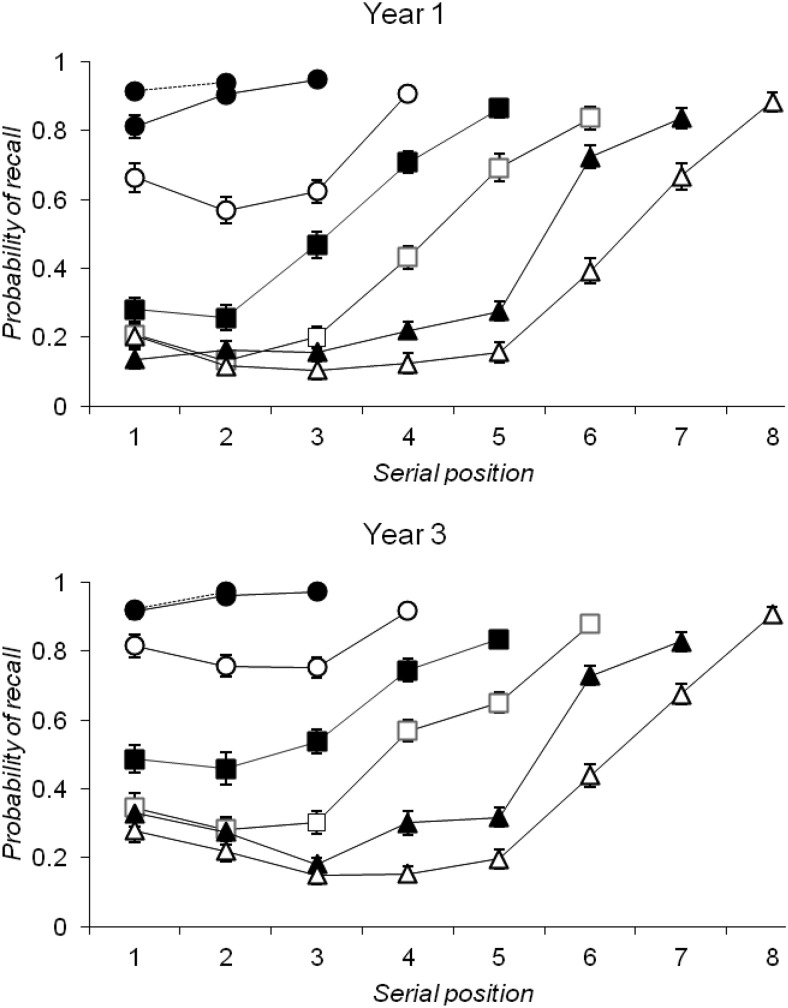
Serial position curves for all list lengths in the free-recall task.

Serial position curves for each year group (see Figure 1) showed a bowing at longer list lengths, with no noticeable serial position effects with less than three and four list items for Year 1 and Year 3, respectively. These data were examined using a series of two-factor mixed ANOVAs, one for each list length, with serial position as a within-subjects factor and year group as a between-subjects factor. There were significant main effects of serial position at all list lengths,^3^ all *p*s < .001, and a significant main effect of year group from list length three onward, all *p*s < .001, which reflected greater levels of recall in the Year 3 group at this, and longer, list lengths. There were significant interactions between serial position and year group on all lists, *p*s < .01, other than at list length two, where a flat serial position curve was observed in both year groups, and at list length eight. On all list lengths between two and eight, there was improved recall of list final items for Year 3 children when compared to Year 1 children, *p*s < .01, and the effect of serial position was significantly greater in Year 1 than in Year 3 individuals. [Fig-anchor fig1]

In addition, there was a decreasing likelihood of participants beginning recall from serial position 1 as lists lengthened. These “probability of first recall of the first item” data were analyzed by a two-factor mixed ANOVA with list length as a within-subjects factor (seven levels) and year group as a between-subjects factor (two levels). There was a significant effect of list length, *F*(6, 594) = 306.342, *p* < .001, *MSE* = 0.035, η_*p*_^*2*^ = .756, which reflected a greater likelihood of starting from the start on list lengths of five and less than on longer lists, *p*s < .05, and a significant main effect of year group, *F*(1, 99) = 10.523, *p* = .002, *MSE* = 0.201, η_*p*_^*2*^ = .096, which reflected a greater likelihood of Year 3 children starting from the start in general. There was also a significant interaction between list length and year group, *F*(6, 594) = 2.603, *p* = .017, *MSE* = 0.035, η_*p*_^*2*^ = .026, which reflected a higher probability of starting from the start in Year 3 than in Year 1 children at all list lengths, *p*s < .05, apart from list lengths two and eight, *p*s > .05.

**Figure 2 fig2:**
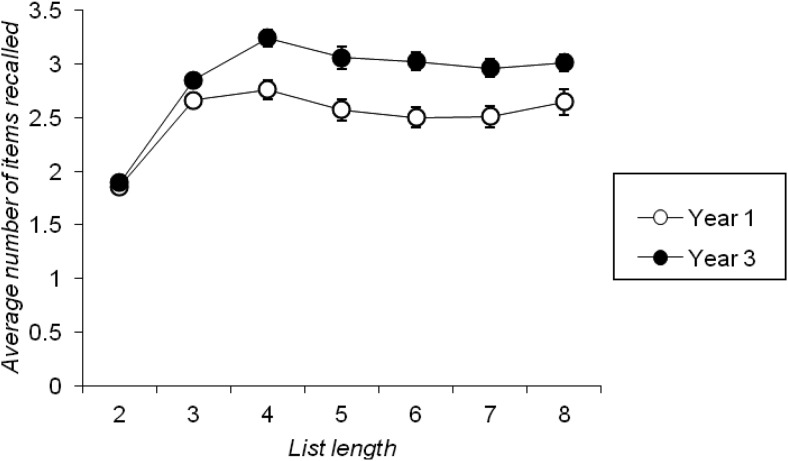
Average number of items recalled in the free-recall task.

Figure 2 plots the average number of items recalled per trial as a function of list length for each year group. A two-factor mixed ANOVA of these data with year group as a between-subjects factor and list length (seven levels) as a within-subjects factor, revealed a significant main effect of list length, *F*(6, 594) = 71.947, *p* < .001, *MSE* = 0.190, η_*p*_^*2*^ = .421, which reflected an increase in the average number of items recalled at list length four when compared to list length two and three, *ps* < 0.01, but no significant difference between the number of items recalled across list lengths five to eight, *p*s > .05. Figure 2 clearly shows that, despite list length increasing, once list length was sufficient for children to recall a set number of items, performance leveled off to a constant value. [Fig-anchor fig2]

This analysis also revealed a significant main effect of year group, *F*(1, 99) = 19.138, *p* < .001, *MSE* = 1.211, η_*p*_^*2*^ = .162, as Year 3 children recalled more items on average than Year 1 children. This effect interacted significantly with list length, *F*(6, 594) = 4.429, *p* < .001, *MSE* = 0.190, η_*p*_^*2*^ = .043, but this was the result of Year 1 children’s average number of items recalled increasing until list length three, and Year 3 children’s average total increasing until list length four. In line with this, a subsequent analysis that examined total recall across list lengths five to eight revealed a nonsignificant main effect of list length, *F*(3, 297) = 1.080, *p* = .358, *MSE* = 0.192, η_*p*_^*2*^ = .011, a significant main effect of year group, *F*(1, 99) = 16.203, *p* < .001, *MSE* = 1.306, η_*p*_^*2*^ = .141, and nonsignificant interaction between these factors, *F*(3, 297) = 0.609, *p* = .609, *MSE* = 0.192, η_*p*_^*2*^ = .006. Individual estimates of capacity for use in subsequent analyses were therefore derived by averaging the total number of items recalled by each individual across list lengths five to eight.

#### Interleaved lists task

**Figure 3 fig3:**
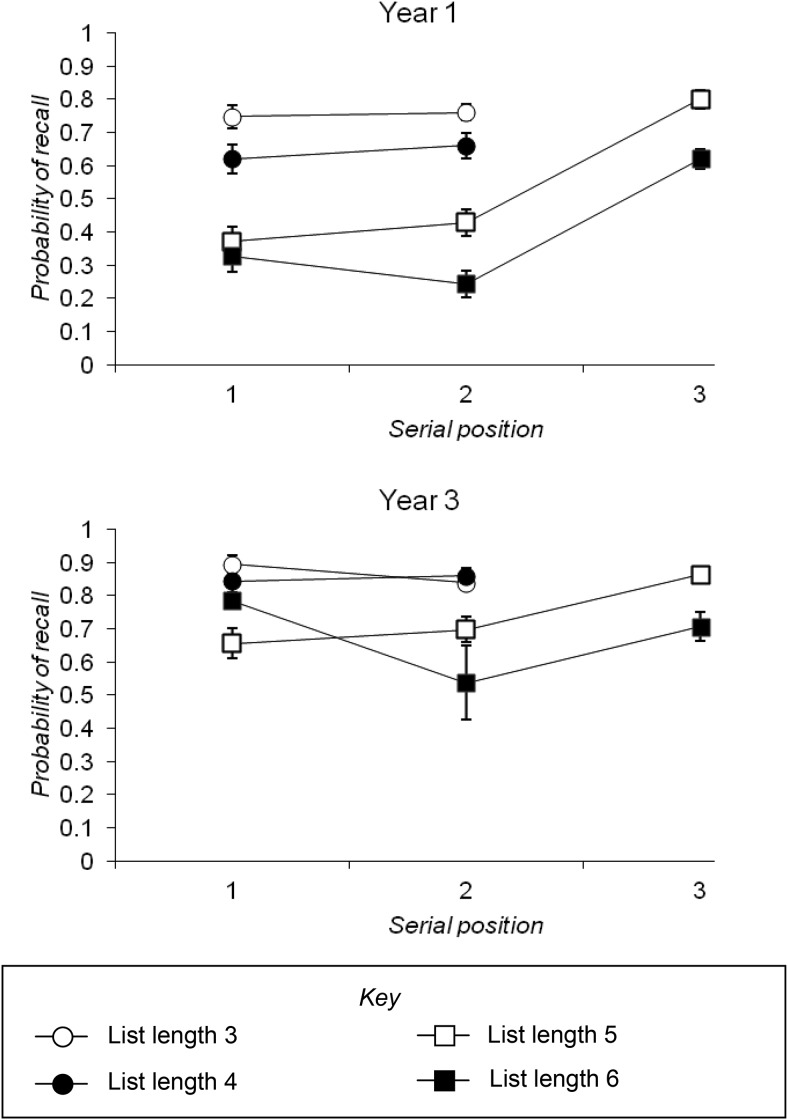
Probability of recall of focal list items in the interleaved lists task, used in Experiment 3, in (top) Year 1 and (bottom) Year 3.

To examine whether focal items were recalled with a “flat” serial position curve, as was anticipated, a 2 × 2 × 2 (for list lengths three and four, with consequent recall of two focal items) or 2 × 2 × 3 (for list lengths five and six, with consequent recall of three focal items) mixed ANOVA was conducted, with year group as a between-subjects factor and list length and serial position as the within-subjects factors (an analysis of developmental changes in total recall follows below). At list lengths three and four, there was no significant main effect of serial position, indicating flatness of the serial position curve, *F*(1, 99) = 0.024, *p* = .876, *MSE* = 0.042, η_*p*_^*2*^ < .001. There was no significant interaction between serial position and year group, as both groups had similarly flat serial position curves for the two attended items at these total list lengths, *F*(1, 99) = 1.245, *p* = .267, *MSE* = 0.042, η_*p*_^*2*^ = .012, and the three-way interaction was also nonsignificant, *F*(1, 99) = 0.458, *p* = .500, *MSE* = 0.025, η_*p*_^*2*^ = .005. At list lengths five and six, there was a significant main effect of serial position, with list-final items being recalled better than earlier list items, *F*(2, 198) = 36.190, *p* < .001, *MSE* = 0.113, η_*p*_^*2*^ = 268. The interaction between serial position and year group was significant, *F*(2, 198) = 10.267, *p* < .001, *MSE* = 0.113, η_*p*_^*2*^ = .040; although there was a significant effect of serial position among Year 3 individuals, *F*(2, 100) = 4.875, *p* = .010, *MSE* = 0.148, η_*p*_^*2*^ = .089, this effect was much more marked among Year 1 children, *F*(2, 98) = 57.873, *p* < .001, *MSE* = 0.078, η_*p*_^*2*^ = .542. Evidence of this can be observed in Figure 3, which plots average recall of the attended item at each serial position by list length for each year group. The three-way interaction in this analysis was not significant, *F*(2, 198) = 1.076, *p* = .343, *MSE* = 0.088, η_*p*_^*2*^ = .011. [Fig-anchor fig3]

The average total number of focal items recalled at each list length was then examined, using a 2 × 4 ANOVA with year group as a between-subjects factor and list length as a within-subjects factor. This revealed significant main effects of both year group, *F*(1, 99) = 40.259, *p* < .001, *MSE* = 0.690, η_*p*_^*2*^ = .289, with Year 3 children recalling more on average than Year 1 children, and list length, *F*(3, 297) = 17.392, *p* < .001, *MSE* = 0.182, η_*p*_^*2*^ = .149. These main effects were qualified by a significant interaction between list length and year group, *F*(3, 297) = 9.479, *p* < .001, *MSE* = 0.182, η_*p*_^*2*^ = .087. The interaction was a reflection of a significant difference in the total number of focal items recalled at each list length among Year 1 children, *p*s < .001, but no significant difference in total recall between list lengths three and four and between list lengths five and six in Year 3 individuals, *p*s > .05; there was a significant difference in total recall between list lengths four and five in Year 3 children. In other words, Year 3 children unsurprisingly recalled more focal items when three as opposed to two attended items were presented, but showed no reliable effect of number of distracters on their recall performance. In contrast, when presented with either two or three focal items, Year 1 children showed an effect of number of nonfocal items in the list.

Analysis of the probability of beginning recall with the first focal item on the just-presented list, with a 2 × 4 ANOVA with year group as a between-subjects factor and list length as a within-subjects factor, revealed a significant main effect of year group, *F*(1, 99) = 36.520, *p* < .001, *MSE* = 0.260, η_*p*_^*2*^ = .269; Year 3 children were significantly more likely to start from the start of the list than were Year 1 children. The main effect of list length was significant, *F*(3, 297) = 64.895, *p* < .001, *MSE* = 0.042, η_*p*_^*2*^ = .396, due to a decreasing likelihood of starting recall with the first focal item with increasing list length, but did not interact significantly with year group, *F*(3, 297) = 1.339, *p* = .262, *MSE* = 0.042, η_*p*_^*2*^ = .013.

#### Split span task

Our pilot work indicated that an individual’s ability to recall Set B when this set was probed first was the most likely indicator of primary memory capacity in this task. Indeed, when examining the average number of items recalled in the current experiment using a 2 × 2 × 2 mixed ANOVA, with year group as a between-subjects factor, and Set (A or B), and recall mode (first or second) as within-subjects factors, a significant main effect of set emerged, *F*(1, 98) = 342.368, *p* < .001, *MSE* = 0.092, η_*p*_^*2*^ = .777; Set B items (*M* = 1.209, *SD* = 0.310) were better recalled than Set A items (*M* = 0.649, *SD* = 0.374) overall. The main effect of recall mode was also significant, *F*(1, 98) = 710.072, *p* < .001, *MSE* = 0.055, η_*p*_^*2*^ = .879, as items recalled first (*M* = 1.241, *SD* = 0.373) were better recalled than items recalled second (*M* = 0.617, *SD* = 0.286). Crucially, there was also a significant interaction between set and recall mode, *F*(1, 98) = 11.480, *p* < .001, *MSE* = 0.047, η_*p*_^*2*^ = .683, which was a result of the mean difference between items recalled first and second in Set B (*M* = 0.941, *SD* = 0.355) being larger than the mean difference between items recalled first and second in Set A (*M* = 0.307, *SD* = 0.300). These analyses confirm that Set B, rather than Set A, items benefitted particularly from being recalled first as opposed to second, implying that it is appropriate to use recall of Set B items when probed first as an indicator of primary memory capacity in this experiment.

**Figure 4 fig4:**
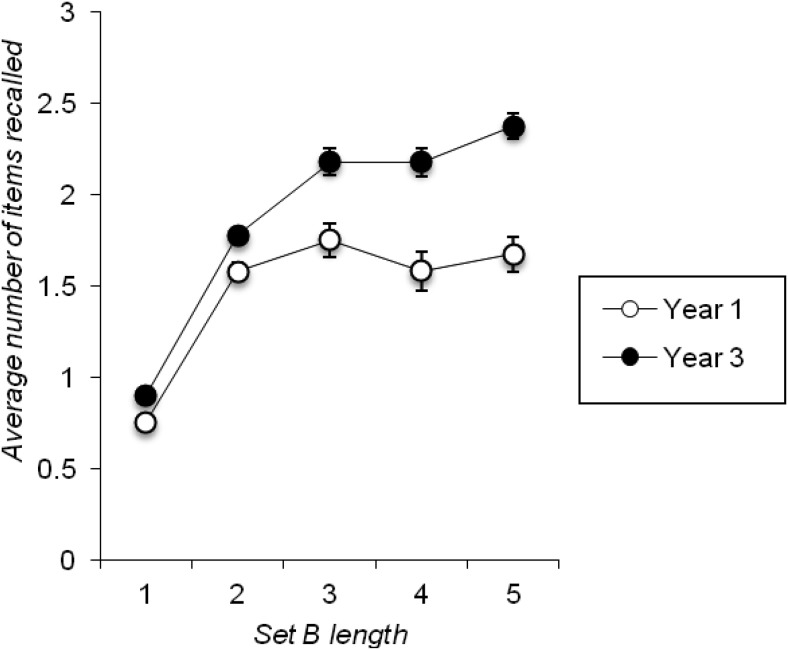
Average number of Set B items recalled on the split span task when this set was probed first.

To examine whether age impacted on recall of Set B (when recalled first), a 2 × 5 mixed ANOVA was conducted with year group as a between-subjects factor and set length as a within-subjects factor (either one, two, three, four, or five items in Set B), with the average number of items recalled in Set B when recalled first as the dependent variable. The results of this analysis are graphed in Figure 4, which clearly shows the significant main effect of year group, *F*(1, 98) = 33.930, *p* < .001, *MSE* = 0.637, η_*p*_^*2*^ = .257, which was a result of Year 3 children recalling more on average than Year 1 children. The main effect of set length was also significant, *F*(4, 392) = 153.996, *p* < .001, *MSE* = 0.156, η_*p*_^*2*^ = .611, with performance at list lengths three, four, and five being better than performance at set lengths one and two, due to ceiling effects on the two shorter list lengths. However, these main effects were qualified by a significant interaction between year group and set length, *F*(4, 392) = 9.531, *p* < .001, *MSE* = 0.156, η_*p*_^*2*^ = .089, as the difference in recall between the two groups was not significant at set lengths 1 and 2, *p*s >.05, but was significant at set lengths 3, 4 and 5, *p*s < .05. [Fig-anchor fig4]

Taken together, these data are clearly in line with the notion of a fixed capacity difference between children in Years 1 and 3 that simply cannot be observed on shorter sets due to ceiling effects. However, a further analysis that examined total recall across set lengths 3 to 5 revealed a main effect of list length that remained significant, *F*(2, 196) = 4.266, *p* = .015, *MSE* = 0.152, η_*p*_^*2*^ = .042, and which still showed a trend toward a reliable interaction with group, *F*(2, 196) = 2.622, *p* = .075, *MSE* = 0.152, η_*p*_^*2*^ = .026. Consequently, and unlike the corresponding analysis for the free-recall task, it appears that longer list lengths would be needed to observe a full flattening off of the number of items recalled from Set B items when these are recalled first.

Consistent with the suggestions that primary memory capacity is taxed by the initial recall of Set B, and that this capacity increases with age, Year 3 children were more likely to begin their recall with the first item in Set B when this set was probed first. Analysis of these probability of first recall data with year group as a between-subjects factor and set length of Set B as a within-subjects factor revealed a significant main effect of year group, *F*(1, 98) = 21.817, *p* < .001, *MSE* = 0.086, η_*p*_^*2*^ = .182, and a significant main effect of set length, *F*(4, 392) = 289.942, *p* < .001, *MSE* = 0.037, η_*p*_^*2*^ = .747, that were qualified by a significant interaction between factors, *F*(4, 392) = 2.985, *p* = .019, *MSE* = 0.037, η_*p*_^*2*^ = .030. The probability of starting recall with the first item in the list decreased with increasing list length, and Year 3 individuals were significantly more likely to begin recall with item 1 than Year 1 children on the list at all set lengths, *p* < .05, with the exception of set length 5, *p* = .398.

### Relative Task Difficulty

A further analysis examined the relative difficulty of the new potential measures of primary memory and simple span, in order to directly compare the average number of items recalled in each task. Because these tasks all included at least some trials that required recall of three items, we compared recall from simple span trials of list length three, recall from free-recall trials of list length three, recall of the three focal items from list length six of the interleaved lists task, and recall of Set B items in the 3:3 condition of the split span task when Set B was recalled first. In order to maximize the comparability of these measures, this analysis scored performance on the simple span task using a free-recall scoring method.

**Figure 5 fig5:**
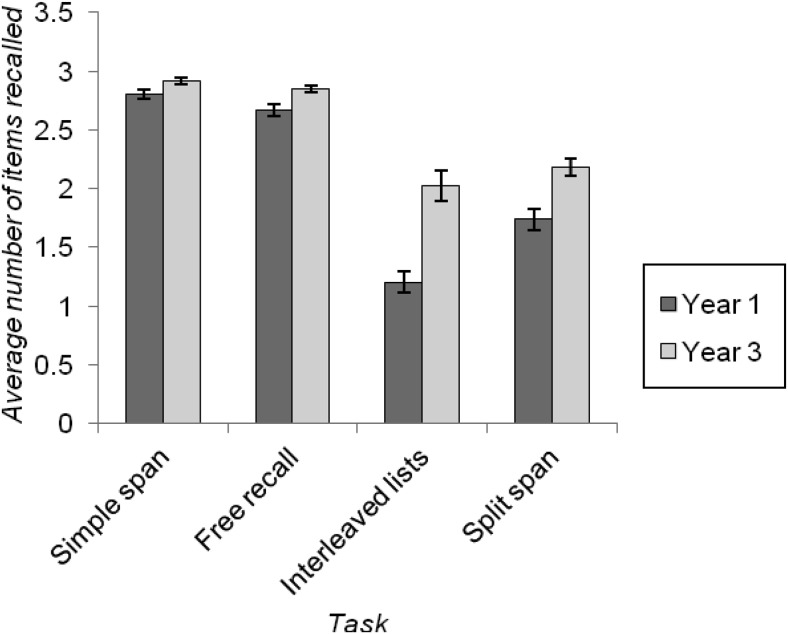
Comparing mean number of items recalled across the four potential tests of primary memory capacity on trials on which three memory items were presented.

Figure 5 plots mean recall by group across these selected conditions of these four tasks. A 2 × 4 mixed ANOVA was conducted on these data. This produced significant main effects of year group, *F*(1, 98) = 36.671, *p* < .001, *MSE* = 0.417, η_*p*_^*2*^ = .272, and task, *F*(3, 294) = 155.186, *p* < .001, *MSE* = 0.238, η_*p*_^*2*^ = .238, that were qualified by a significant interaction between these factors, *F*(3, 294) = 10.802, *p* < .001, *MSE* = 0.238, η_*p*_^*2*^ = .099. The main effect of task reflected the fact that the average number of items recalled in the simple span task (with free-recall scoring) was significantly greater than that in the free-recall task; in turn, free-recall performance was significantly higher than that seen on the split span task, which itself gave rise to significantly superior recall than the interleaved list task, *p*s < .001. Although the effect of year group was significant for each task, the interaction was driven by larger year group effects on the interleaved lists task, *F*(1, 98) = 26.220, *p* < .001, *MSE* = 0.646, η_*p*_^*2*^ = .211, and the split span task, *F*(1, 98) = 14.030, *p* < .001, *MSE* = 0.346, η_*p*_^*2*^ = .125, than on the free-recall task, *F*(1, 98) = 9.404, *p* = .003, *MSE* = 0.092, η_*p*_^*2*^ = .088, or the simple span task, *F*(1, 98) = 6.967, *p* = .010, *MSE* = 0.046, η_*p*_^*2*^ = .066. Furthermore, while the size of the group effect across the simple span and free-recall task was broadly comparable as assessed by the interaction of year group and task across these two tasks, *F*(1, 98) = 1.660, *p* = .201, *MSE* = 0.039, η_*p*_^*2*^ = .017, the magnitude of the group effect was significantly larger in the split span task than in the free-recall task, *F*(1, 98) = 5.739, *p* = .018, *MSE* = 0.142, η_*p*_^*2*^ = .055, and, in turn, was significantly larger in the interleaved lists task than in the split span task, *F*(1, 98) = 4.286, *p* = .041, *MSE* = 0.426, η_*p*_^*2*^ = .042.[Fig-anchor fig5]

### Individual Differences Analyses

One of the aims of the current study was to create new estimates of primary memory capacity. In order to determine whether the average number of items recalled on a given task was related to probability of first recall of the first item in a list on that task, bivariate correlations between these two types of measure were examined. Within all tasks and in each age group, probability of first recall of the first item on a list was moderately to highly correlated with average number of items recalled, (*r*s between .882 and .311, all *p*s < .05). Children who recalled more items on average were therefore more likely to start recall from the start of the list.

In order to examine how the new measures of primary memory were related to simple and complex span performance, and to measures of academic attainment, correlations and linear regression modeling were used. Preliminary analyses indicated that the associations with these predictors were broadly similar when using either the average number of items recalled or the probability of beginning recall with the first item on the list. For this reason, and given the reliable correlations between these two indices of primary memory that were drawn from each of our novel measures (see earlier), only the average number of items recalled on these new tasks was included in the individual differences analyses that follow. A correlation matrix showing the associations between this measure from each novel task, simple and complex spans, and reading and mathematics performance, is presented in Table 2.[Table-anchor tbl2]

### How Do the New Memory Measures Relate to Performance on Simple Span and Complex Span Tasks?

**Figure 6 fig6:**
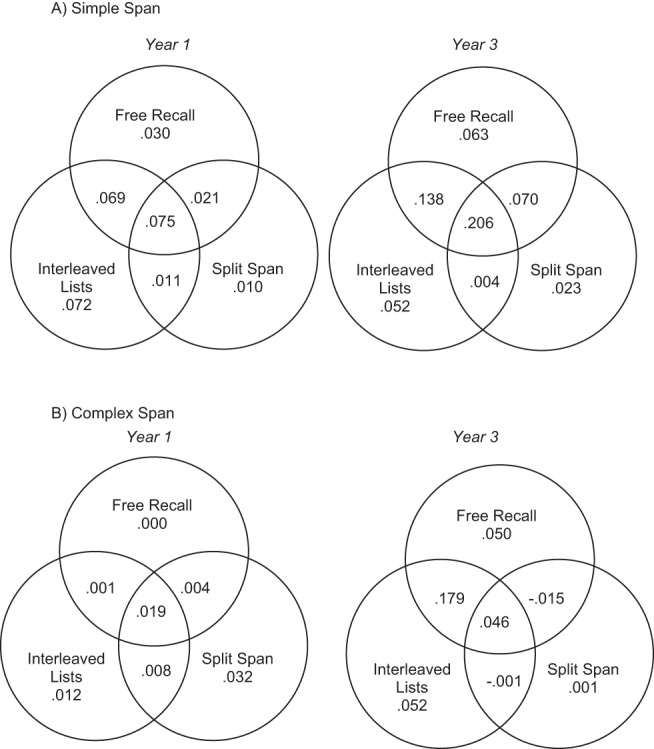
The unique and shared variance contributed to the prediction of span measures by average number of items recalled in each of the novel measures. Panel A shows variance predicted in simple span, and Panel B shows variance predicted in complex span.

Table 2 shows that simple span was significantly correlated with all of the novel memory measures in both Year 1 and Year 3 children, but complex span was only related to the novel measures in Year 3 children. In order to unpick the relationships between the memory measures further, linear regression modeling was used to partition the variance in simple and complex span and to examine relationships between each new measure and these two more traditional memory span indices. By using this method, the commonalities between the measures, and unique contributions each measure makes, in predicting simple and complex span can be determined (see, e.g., Salthouse, 1994). Venn diagrams showing shared and unique variance for the prediction of simple span are shown in Figure 6 (Panel A), separately for each year group, showing the relative variance in simple span predicted by the average number of items recalled from each of the novel measures.[Fig-anchor fig6]

A significant proportion of variance in simple span was predicted by recall accuracy on the novel measures in both Year 1 children, *r*^2^ = .288, *F*(3, 39) = 5.262, *p* = .004, and in Year 3 children, *r*^2^ = .556, *F*(3, 45) = 18.754, *p* < .001. The largest amount of unique variance predicted by the novel tasks came from the interleaved lists task for both age groups (see Figure 6, Panel A). While this task shared a small (and nonsignificant) amount of variance with free recall in the Year 1 children, in the Year 3 children there was a significant amount of shared variance between the interleaved lists and free-recall task, which suggests that both tasks are measuring a similar construct. This analysis suggests that the mechanisms underpinning simple span are shared with the novel tasks, and that this shared variance may indeed reflect the primary memory contribution to simple span, particularly in Year 3 children.

In contrast, the new measures predicted smaller amounts of variance in complex span, see Figure 6 (Panel B). Here, recall scores did not predict a significant amount of variance in Year 1 children’s complex span performance, *r*^2^ = .076, *F*(3, 39) = 1.071, *p* = .372, but did predict significant variance in the Year 3 group, *r*^2^ = .312, *F*(3, 45) = 6.788, *p* = .001. Although the novel tasks therefore did predict significant amounts of variance in complex span in the Year 3 children, the amounts were smaller than the variance in simple span predicted by the novel tasks in both age groups.

A series of stepwise regressions was then used to examine whether performance on the novel tasks contributed anything to the prediction of complex span performance, over and above that predicted by simple span. Separate models were examined for each age group, but in each case, simple span partial credit score was entered on the first step. Simple span accounted for a significant proportion of variance in complex span in both Year 1, *r*^2^ = .094, *F*(1, 41) = 4.240, *p* = .046, and Year 3, *r*^2^ = .198, *F*(1, 47) = 11.587, *p* = .001, children. Then, on the second step of each regression, average number of items recalled from either free recall, the interleaved lists task, or the split span task was entered. A summary of the results of the second step of each of these regressions is presented in Table 3.[Table-anchor tbl3]

Table 3 shows that in Year 1, none of the measures taken from the novel tasks predicted complex span to a significant degree once simple span was accounted for. However, among Year 3 children, average number of items recalled on the free-recall task and interleaved lists task contributed significant proportions of variance to the prediction of complex span when simple span was first accounted for.

### How Well Do the Novel Tasks Predict Reading and Mathematics?

**Figure 7 fig7:**
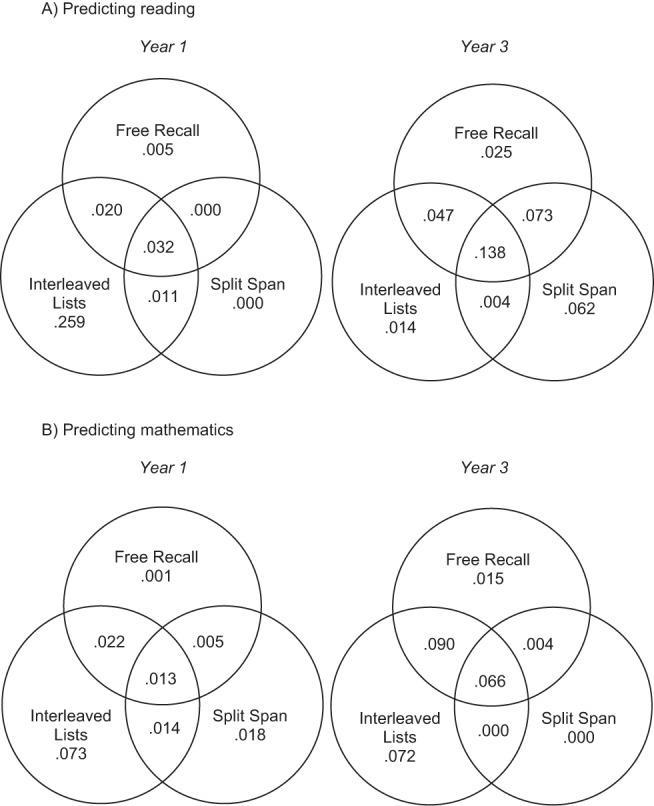
The unique and shared variance contributed to the prediction of academic measures by the average number of items recalled on the novel measures. Panel A shows variance predicted in reading; Panel B shows variance predicted in mathematics.

A series of linear regression models tested how well recall accuracy on the novel tasks predicted variance in reading and mathematics. Partitioned variance from these analyses is shown in Figure 7. Overall, these models predicted significant variance in reading among Year 1 children, *r*^2^ = .327, *F*(3, 39) = 6.316, *p* = .001, and Year 3 children, *r*^2^ = .363, *F*(3, 45) = 8.561, *p* < .001. Among Year 1 pupils, the interleaved lists task contributed the majority of variance to the prediction of reading with no significant shared variance between free recall and split span. In Year 3 children, shared variance between the three tasks contributed the majority of significant variance to the prediction of reading. Performance on the novel tasks also predicted significant variance in mathematics in Year 1 individuals, *r*^2^ = .180, *F*(3, 39) = 2.858, *p* = .049, and in Year 3 pupils, *r*^2^ = .247, *F*(3, 45) = 4.915, *p* = .005. In both Year 1 and Year 3 groups, the interleaved lists task was the strongest unique predictor of mathematics performance but once again there was more shared predictive variance between the three novel tasks in Year 3 individuals.[Fig-anchor fig7]

In order to determine whether the average number of items recalled in the novel tasks predicted any variance in reading and mathematics above that predicted by simple span alone, a series of stepwise regressions were then conducted. These were carried out separately for each measure of academic attainment and each group, but always entered simple span partial credit score on the first step. Then, on the second step of each regression average number of items recalled on either free recall, the interleaved lists task, or the split span task was entered. Summary statistics for the results of the second steps of these regressions are presented in Table 4. These show that in Year 1 children, average recall on the interleaved lists task contributed significant variance to the prediction of reading over and beyond that contributed by simple span. In Year 3, free recall and split span recall contributed significant extra variance to the prediction of reading. The interleaved lists task was the only task to contribute significant additional variance to the prediction of mathematics over and above simple span, and this was true of both Year 1 and Year 3 groups.[Table-anchor tbl4]

## Discussion

This experiment was conducted with three main objectives. The first was to explore novel ways of measuring primary memory capacity to determine whether this construct can be properly assessed in children; this was done by exploring specific recall characteristics in three new tasks. The second was to investigate developmental change in these primary memory indices. The third was to determine whether these novel measures of primary memory capacity were better predictors of working memory and academic attainment than a standard test of ISR.

### Can We Measure Children’s Primary Memory From Free Recall, the Interleaved Lists Task, and the Split Span Task?

This study did not restrict the measurement of primary memory to ISR as assessed using span tasks, and went beyond estimating primary memory solely from free-recall performance (e.g., Gibson et al., 2013; Unsworth & Engle, 2007; Unsworth, Spillers, & Brewer, 2010). Although our three new measures led to different levels of performance (see Figure 5), showing that they are not all pure measures of a single construct, the findings support the view that they do all index primary memory capacity to a meaningful extent. In particular, as the number of items in the to-be-remembered set increased, there came a point at which serial position curves began to bow. Of course, this is entirely unsurprising and reflects the fact that a longer memory list is less likely to be recalled successfully. However, the key point is that the extent to which serial position curves were flat interacted with the recall demands of the task. So, for example, in the split span task, relatively flat serial position curves were observed for shorter Set B (the second present subset of items) lists, but only when the trial probed these items first.

In addition, as list lengths increased there was some evidence that the number of items recalled from a trial reached a fixed capacity level (similar to the level observed in Cowan et al.’s [e.g., Cowan, Elliott et al., 2005; Cowan, AuBuchon et al., 2011] focus of attention studies). This was particularly apparent for the free-recall task (see Figure 2), but was also seen to some extent in the number of Set B items recalled when that set was probed first in the split span task (see Figure 4). It should be noted that this evidence of a constant recall capacity that is independent of the total number of items presented on the list may well be indicative of a limited but fixed storage capacity which is affected by output interference (see, e.g., Murdock, 1968; Lewandowsky, Duncan, & Brown, 2004; Oberauer, 2003), rather than being a direct reflection of a store with a predetermined number of “slots” (cf. Broadbent, 1958). While the requirement to verbally output the memoranda in these tasks may well lead to an estimate of capacity that is somewhat smaller than the number of items an individual can maintain without outputting them, this does not undermine our measures as potential indices of individual differences in primary memory capacity.

Another point to note is that the two key indices that were extracted from each of the three new paradigms were highly correlated within a given task. These were the average number of items recalled from all trials (other than in free recall, where list lengths five through eight were used), and the probability of first recall of the first occurring (or probed) item (as a signifier of an individual attempting to recall in forward serial order). The significant correlations between the two measures for each task indicates that children recalling a higher average number of items were also more likely to attempt recall in serial order by beginning at the start of the list. This provides further support for the claim that spontaneous serial ordering at recall reflects the use of primary memory (cf. Broadbent, 1958; Penney, 1989; Sperling, 1967). Most importantly, regression analysis of performance across all measures showed that the three new tasks successfully predicted significant portions of variance in simple span, and that there was substantial shared variance between them in this prediction. As Figure 6 shows, among Year 1 children, the variance shared between capacity estimates derived from the interleaved lists task and the free-recall task predicted 14.4% of the variance in simple span. Among Year 3 children, the corresponding value was 34.4%, with 20.6% of this variance being shared by all three of the new measures. While it would be incorrect to say that the new measures are therefore collinear with simple span performance, this may reflect the fact that simple span itself is not a pure measure of primary memory capacity. The total variance in simple span predicted by the three primary memory measures was only 28.8% (Year 1) and 55.6% (Year 3), and this may well reflect the impurity of performance on a simple span task, which we suggest is a combination of primary memory, secondary memory, and strategy use such as rehearsal.

We propose that, as the definition of primary memory specifies the processes and characteristics of immediate memory recall, focusing on the number of items within the current focus of attention and spontaneous serial recall, the interleaved lists, split span, and free-recall tasks are better placed to accurately estimate primary memory capacity than is simple span. Indeed, if the STM model were used as the explanatory framework from which to estimate capacity from these tasks (with the only characteristic being duration of the recall period), every item recalled in each task would be assumed to be held within immediate memory. By focusing instead on *process,* as we do in the current study, a distinction can be drawn between the items within a recalled set that are held in primary memory and any additional items held in secondary memory.

### Is There Developmental Change in Primary Memory Estimates?

In all three novel tasks a developmental increase in the number of items successfully recalled between Year 1 and Year 3 individuals was observed. For example, the serial position curves for probed items in the interleaved lists task revealed flatter serial position curves at relatively longer list lengths in the older group, as shown in Figure 3. In common with Parkinson’s (1974) finding that adults with larger digit spans were more able to remember more items from both an “attended” and “unattended” stream, the direct comparison between Year 1 and Year 3 children in this experiment showed that older children with, on average, larger capacities in the simple span task were also able to recall more items from a focal stream in the interleaved lists task. Children in Year 1 showed marked recency for the end list items in the three-item lists of the interleaved lists task, while children in Year 3 were more likely to show a flattened serial position curve at this list length.

As further support for development in capacity, the free-recall task provided evidence that developmental populations demonstrate the same patterns of serial recall and probability of first recall as observed in adults by Ward et al. (2010), but at considerably reduced list lengths. Notably, serial position curves exhibited bowing on three-item lists in Year 1 children, and on four-item lists in Year 3 children (see Figure 1). The split span task employed the same list lengths for each age group in order to allow a direct comparison of performance between year groups. However, once set lists were long enough to exceed younger children’s recall capacity for Set B items when this set was probed first, stable capacity differences emerged across the years that echoed those seen in the free-recall task.

This age effect was also seen in the direct comparison of performance across tasks when three to-be-remembered items were presented (see Figure 5), which, in turn, shows that estimates of primary capacity for verbal material that is verbally recalled extend no further than around two items in 6-year-olds, and no further than around three items in 8-year-olds (see also Figure 2 and Figure 4). Furthermore, similar developmental differences were seen on our other key index of primary memory capacity, namely individuals’ likelihood of beginning recall with the first presented to-be-remembered item (see also Dempster, 1981). These findings imply that children’s primary memory capacity improves with age. This claim contrasts with previous suggestions from the free-recall literature (Cole, Frankel, & Sharp, 1971; De Alwis et al., 2009; Thurm & Glanzer, 1971). It also implies that developmental change in tasks such as complex span that potentially tap both primary and secondary memory (Unsworth & Engle, 2006) could be driven by age-related improvements in primary memory capacity. This highlights the importance of designing appropriate measures of this construct, which go beyond the constraints imposed by traditional span procedures, in order to properly understand the causes and consequences of its development.

### How Do the Novel Measures of Primary Memory Relate to Academic Performance?

It is important to note that the same reading test was given to all children while mathematics performance was examined using age-appropriate tests. Among Year 3 children, both reading and mathematics exhibited a normal distribution of performance. However, in the Year 1 children, although reading performance was normally distributed, the mathematics test appeared to be rather too easy, resulting in a skewed distribution. Any analysis of the mathematics test in the Year 1 children should, therefore, be treated with a degree of caution. The regression models for mathematics performance in Year 1 predicted smaller amounts of variance than those for the Year 3 group, and this may well be an artifact of the limited range of scores in the test among younger individuals. Furthermore, all of the memory tests in the current paper were verbal, which rules out any potential analysis of the contribution of visual immediate memory to reading and mathematics in this particular study.

With this in mind, the average number of items recalled in the three new tasks predicted significant portions of variance in reading (see Figure 7), with the interleaved lists task contributing the most unique variance to reading in the Year 1 children. In the Year 3 children, shared variance between the three new primary memory measures contributed the most variance to the prediction of reading. Furthermore, the interleaved lists task contributed a significant portion of variance to performance in reading even when simple span was taken into account in the Year 1 children (see Table 3). Among the Year 3 children, this was not the case, and taking simple span into account resulted in only the split span task having additional predictive value. This reinforces the idea that the novel tasks developed in this set of experiments are measuring a related capacity to that derived from simple span. Performance on the novel measures was not as successful in predicting mathematics performance, but nevertheless there were similar relationships between the new variables and mathematics. Most notably, the interleaved lists task was the greatest sole predictor of performance, and, in this instance, this was true in both Year 1 and Year 3 groups, and even when simple span performance was first taken into account.

In contrast to the predictive value of the recall indices derived from the novel tasks and from simple span on academic achievement, complex span was not a good predictor of academic performance. The absence of a strong relationship between complex span and academic attainment in the Year 1 children may, in part, at least, reflect floor effects on the former measure. A further potential issue with this particular complex span task is that it may not have indexed skills that are deemed important to the association between working memory and achievement. Specifically, the color naming task used as the processing activity in the current complex span task was designed to fully fill each processing period. Other complex span tasks require participants to perform a single processing operation within a fixed time window and consequently provide potential opportunities for rehearsal or other maintenance-related activities (Jarrold & Bayliss, 2007). Individual differences in the use of such maintenance-related strategies may therefore have been obscured by the processing requirements of the current complex span task, limiting its predictive power.

While the current study does provide evidence to suggest that primary memory capacity is a reliable predictor of academic attainment, the novel and traditional measures of memory used in the current study certainly do not predict all of the variance in reading, and predict only a small portion of variance in mathematics. Furthermore, as discussed earlier, the value of the complex span task as a predictive measure of academic achievement may have been limited. We would not want to suggest, therefore, that primary memory is a better predictor of achievement than is working memory. Rather, the important point is that the primary memory measures developed in this paper were better predictors of reading (in Year 1) and of mathematics (in both age groups) than was a traditional simple span task. This adds further weight to our initial claim that it is critical to measure primary memory carefully, in combination with other likely contributors to working memory performance (such as speed of processing, Christopher et al., 2012; and management of distraction, Hale, Bronik, & Fry, 1997) to determine the relative contributions of these skills and abilities to academic performance.

Another point to note is that while there was considerable shared predictive variance between the three novel measures in the prediction of reading abilities in Year 3 individuals, this was not the case for children in Year 1 (see Figure 7). Instead, among Year 1 individuals, the interleaved lists task was a unique contributor to the prediction of variance in reading. There are elements of the interleaved lists task that are particularly intriguing from an educational perspective. Specifically, in this paradigm children are required to selectively attend to one character who is deemed to be more important than another. The similarities between the structure of this task and typical classroom demands (focusing on a teacher and ignoring distracting information) make it particularly interesting in the context of understanding the link between storage capacity and educational attainment.

The interleaved lists task was developed as a primary memory measure (after Bryden, 1971; Broadbent, 1958, and our own pilot work), and shared variance with the other two novel tasks. Similarly, a number of tasks which have been used to index the focus of attention in other work also require inhibition of irrelevant or distracter items (e.g., Cowan, Elliott et al., 2005, Cowan, AuBuchon et al., 2011). It is therefore established that the presence of irrelevant items does not necessarily prevent a task from being a valid measure of immediate, or in the current case, primary memory. Nevertheless, the interleaved lists tasks clearly does have in common with working memory tasks the need for interference control (items to-be-remembered are interleaved with items to-be-ignored). As such, it may also index executive abilities to some degree (cf., Swanson & Cochran, 1991), although the relative importance of this constraint may well vary with age and ability.

This may, therefore, account for the strong association between the interleaved lists task and the academic measures, particularly among Year 1 children. The analysis of relative levels of task difficulty (see Figure 5) showed that Year 1 children were considerably more affected by the presence of distracters in the interleaved lists task, relative to their level of performance on tasks without distraction, than were the older children. If the Year 1 children were particularly susceptible to the effects of distracters, even when explicitly told to ignore these items, then this may explain why the interleaved lists task was a better predictor of, for example, reading comprehension, than were the other primary memory indices in this younger group.

With this in mind, the interference experienced by the younger children implies that somewhat purer indices of primary memory capacity in children under 7 might be provided by our split span task and our modified version of the free-recall task. The former task is novel, though designed on the basis of the theoretical arguments outlined initially. The latter has been used with adults (e.g., Ward et al., 2010), but here we show the total number of items recalled on this task, across varying list lengths, may provide a better measure of primary memory capacity than other more traditional approaches that have previously been applied to free-recall data (cf. De Alwis et al., 2009; Tulving & Colotla, 1970, see also Jarrold et al., 2013)

## Conclusions

Overall, this experiment has introduced novel ways that have the potential to measure primary memory capacity in developmental populations. By analyzing both recall accuracy and the analysis of probability of first recall on these tasks, reliable estimates of primary memory have been extracted which show clear developmental improvement, countering previous claims that primary memory capacity is age-invariant. This experiment has further shown the importance of individuals’ ability to retain a set amount of information in serial order as a characteristic of primary memory, and in turn, the importance of primary memory contributions to academic achievement. Finally, the interleaved lists task is a novel and reliable measure that emerges as a particularly strong predictor of academic achievement in young children and which has obvious educational relevance. The predictive power of this task may partly follow from the fact that young children find it particularly hard to resist distraction from irrelevant items. If so, then this task would provide a composite measure of primary memory capacity and resistance to distraction in young children that would be analogous to the more traditional complex span task used in adult studies (cf. Kane, Bleckley, Conway, & Engle, 2001; Unsworth & Engle, 2007), but that would be of a much more appropriate level of difficulty for use in future developmental studies.

## Supplementary Material

10.1037/a0039464.supp

## Figures and Tables

**Table 1 tbl1:** Descriptive Statistics for Participants Providing Data on All Measures

	Year	*M*	*SD*	Min.	Max.	Alpha	S	K
Simple span (partial credit score)	1	13.02	2.35	7.00	19.20	.79	−0.26	0.93
	3	16.47	3.38	6.83	23.24	.89	−0.22	0.30
Complex span (partial credit score)	1	4.44	2.50	0.50	10.42	.72	0.48	−0.44
	3	8.43	3.26	3.00	19.51	.81	1.22	2.17
Free recall (average no. recalled from LLs 5−8)	1	2.51	0.51	0.60	3.50	.87	−1.28	3.79
	3	3.01	0.50	1.85	4.05	.79	−0.16	−0.15
Interleaved lists (average no. recalled)	1	1.41	0.39	0.55	2.30	.82	0.06	−0.18
	3	1.90	0.39	0.90	2.45	.66	−1.06	0.54
Split span (average no. recalled)	1	1.47	0.38	0.60	2.04	.91	−0.45	−0.29
	3	1.88	0.32	1.02	2.48	.91	−0.41	0.34
Free recall (p 1st recall of Item 1)	1	0.36	0.12	0.11	0.60	.84	−0.40	−0.41
	3	0.50	0.18	0.06	0.94	.81	0.22	0.49
Interleaved lists (p 1st recall of Item 1)	1	0.40	0.25	0.00	0.95	.85	0.46	−0.79
	3	0.71	0.25	0.10	1.00	.83	−1.11	0.27
Split span (p 1st recall of Item 1)	1	0.33	0.11	0.07	0.68	.52	0.60	1.84
	3	0.46	0.15	0.17	0.80	.64	0.20	−0.07
Reading (total score)	1	17.65	9.88	4	41	.95	0.72	−0.02
	3	30.08	8.63	5	45	.91	−0.85	0.78
Mathematics (total score)	1	22.79	4.59	8	28	.90	−1.65	2.67
	3	22.29	8.28	3	34	.92	−0.42	−0.79
*Note*. S = skewness; K = kurtosis; LLs = list lengths; p = probability.								

**Table 2 tbl2:** Bivariate Correlations Between Memory and Academic Measures

	1	2	3	4	5	6	7
1. Simple span (partial credit score)		.306*	.442**	.476**	.342*	.477**	**.272**
2. Complex span (partial credit score)	.445**		**.155**	**.201**	**.251**	**.183**	**.111**
3. Free recall (average no. recalled from LLs 5–8)	.690**	.509**		.522**	.488**	**.239**	**.274**
4. Interleaved lists (average no. recalled)	.632**	.505**	.657**		.376*	.567**	.395**
5. Split span (average no. recalled)	.550**	.295*	.607**	.435**		**.210**	**.290**
6. Reading (total score)	.499**	.446**	.533**	.451**	.527**		.609**
7. Maths (total score)	.379**	.360*	.418**	.477**	.264	.673**	
*Note*. LL = list lengths. The upper half of the diagonal (boldface) presents correlations for Year 1 children, the lower half for Year 3 children.							
* *p* < .05. ** *p* < .01.							

**Table 3 tbl3:** The Amount of Variance in Complex Span Partial Credit Score, With Simple Span Partial Credit Score Already Controlled for, Predicted in Each Year Group by Performance on Potential Tests of Primary Memory Capacity

Independent variable type	Year	Independent variable entered	Δ *R*^*2*^	*F*	*df*	*p*
Average number of items recalled	1	Free recall	<.001	0.021	1, 40	.885
		Interleaved lists	.004	0.172	1, 40	.680
		Split span	.024	1.095	1, 40	.302
	3	Free recall	.078	4.970	1, 46	.031
		Interleaved lists	.083	5.341	1, 46	.025
		Split span	.004	0.206	1, 46	.652
*Note.* *df* = degrees of freedom.						

**Table 4 tbl4:** The Amount of Variance in Reading and Mathematics, With Simple Span Partial Credit Score Already Controlled for, Predicted in Each Year Group by Average Number of Items Recalled on Potential Tests of Primary Memory Capacity

Dependent variable	Year	Independent variable entered	Δ *R*^*2*^	*F*	*df*	*p*
Reading	1	Free recall	.001	0.050	1, 40	.824
		Interleaved lists	.150	9.608	1, 40	.004
		Split span	.002	0.128	1, 40	.722
	3	Free recall	.068	4.561	1, 46	.038
		Interleaved lists	.031	1.964	1, 46	.168
		Split span	.091	6.367	1, 46	.015
Mathematics	1	Free recall	.029	1.308	1, 40	.260
		Interleaved lists	.091	4.368	1, 40	.043
		Split span	.044	2.002	1, 40	.165
	3	Free recall	.047	2.652	1, 46	.110
		Interleaved lists	.094	5.663	1, 46	.022
		Split span	.004	0.235	1, 46	.630
*Note.* *df* = degrees of freedom.						

## Footnotes

1 It is important to point out that Ward et al. (2010) would reject the notion that their data support a distinction between primary and secondary memory, and would instead subscribe to a more unitary explanation of free recall performance.

2 Using Bonferroni pairwise comparisons here and for all corresponding subsequent analyses, all *p*s smaller than .05.

3 Cronbach’s alpha values for the simple and complex span tasks were derived by calculating separate partial credit scores for each of the first, second, third, fourth, and fifth trial at each list length. However, it should be noted that the stopping rule used for these tasks means that these measures are not independent of one another, which risks inflating the reliability estimate.
