# Supplementary material for: The Developmental Influence of Primary Memory Capacity on Working Memory and Academic Achievement
Source: Dev Psychol. 2015 Jun 15;51(8):1131–47. doi: 10.1037/a0039464 (PMC4536926; doi:10.1037/a0039464)
Supplement: Supplementary file 1 [file z2p006153524sb01.doc]

**Supplemental Materials**

**The Developmental Influence of Primary Memory Capacity on Working Memory and Academic Achievement**

**by D. Hall et al., 2015, *Developmental Psychology***

**http://dx.doi.org/10.1037/a0039464**

Pilot Study 1

*Method*

Twelve children (6 girls, mean age 7 years 0 months, from 6 years 6 months to 7 years 6 months) from one local school participated in this study, for whom parents had provided consent.

*Design*

Eighty-four trials in total were administered, comprising 6 conditions each of 14 trials. These conditions are documented in Table 1, which illustrates how they differed with respect to list length and / or sequence order within the list, the latter relating to whether a non-focal item was a prefix or suffix. In each condition there were also three recall modes: the majority of the trial types requested recall of focal items only, such that the experimental design gave priority to these items. The remaining trials sequentially probed both focal and non-focal items in both possible orders. Trials of each type and from each condition were pseudo-randomly organized within five blocks, so that children would not anticipate list length or recall mode.

*Materials and Procedure*

Children were tested in two sessions lasting approximately 20 minutes each and were introduced to two cartoon characters, SpongeBob and Patrick, who were identified by illustrations and two distinct male voices. The task was presented on an Apple MacBook computer through internal speakers. Words for the task were chosen from a pool of 263 concrete nouns with an age of acquisition of less than 6.2 years (statistics derived from the MRC database, Wilson, 1988). 139 words were spoken by the SpongeBob voice, the remaining 124 words were spoken by Patrick. There were no repeated words between or within trials.

Children were explicitly told to pay attention to SpongeBob (i.e., focal stimuli) and try to remember his words, and to try to ignore Patrick (non-focal items). Whenever a focal item was presented, SpongeBob appeared on the left hand side of the computer screen with a speech bubble in the centre of the screen showing a color illustration of the word for 1,000 ms, concurrent with presentation of an audio recording of the word in a male voice. Similarly, when a non-focal item was presented Patrick appeared on the right hand side of the computer screen with a speech bubble in the centre of the screen showing a color illustration of the word for 1,000 ms, alongside an audio recording of the word in the second male voice.

Following stimulus presentation, if the focal items were probed first SpongeBob’s reappeared with a red speech bubble in the centre of the screen, to cue recall of his words. If nonfocal items were probed, at the relevant probe point Patrick reappeared with a red speech bubble in the centre of the screen. In each recall mode, children were told they could recall any of that set of items (e.g., focal items) in any order, but were not to recall the other set (i.e., the nonfocal items). Where recall of a second set of items was required, recall began immediately after children had indicated they could no longer recall any words from the first probed set.

*Results*

Figure 1 plots recall of both focal and non-focal items, under each recall mode, for each condition of the experiment. A first point to note about these data is that the two conditions in which the trial started with the presentation of a non-focal item (LL4B, LL6B) led to somewhat different patterns of performance than the other four conditions, specifically showing more recency in the recall of focal items (compare the graphs for LL4A and LL4B) and less recency in the recall of non-focal items. In contrast, the conditions in which the list started with a focal item produced serial position curves more in line with those reported by Bryden (1971) and Parkinson (1974). One possibility is children were surprised by the occurrence of a trial employing the less common presentation order, and, given this concern, all analyses reported below focus only on the four conditions employing the more common presentation order (LL3, LL4A, LL5, LL6A).2 In these analyses we address two main aspects of the data. First, whether serial position curves are ‘flat’ for both focal and non-focal elements. Second, whether primary memory capacity estimates can be derived from analysis of the probability of first recall alongside data on total recall.

*Serial position curves at each list length for focal items*

Initially we examined focal set recall. Recall probabilities were compared in a 3 x 2 (for list lengths 3 and 4) or a 3 x 3 (for list lengths 5 and 6) ANOVA. The first factor involved recall mode (focal items alone, focal items recalled first, and focal items recalled second), and the second factor involved serial position of the focal item (2 or 3 recall items). Summary statistics for these analyses are presented in Table 2.

The first key finding is that we obtained a recall mode effect across all list lengths of these four conditions. Subsequent post-hoc analysis confirmed significantly fewer correct recalls when focal items were probed second rather than first.3 There was no significant difference between focal recall alone and focal recall first, as would be expected (all *p*s greater than .05). The second key finding is the equivalence of serial position effects for list lengths 3 and 4 (where there are two focal items), confirming that serial position curve for focal items is statistically flat, with both items equally likely to be recalled. In contrast, there was a significant interaction between recall mode and serial position at list lengths 5 and 6. Recall for serial positions 1 and 2 did not differ in recall alone or recall first versions, *p*s > .10, but there was a significant recency effect (i.e., advantage for position 3 relative to 1 and 2), all *p*s < .01. When the focal items were recalled second, there was no difference between serial positions, *p*s > .05.

*Serial position curves for non-focal items*

We next explored non-focal recall. For this analysis, there were necessarily only two recall modes to consider; recall before or after the focal items. Summary statistics for these analyses are presented in Table 3.

As Table 3 shows, recall probabilities of non-focal items were reliably affected by recall mode; fewer items were remembered when probed second, all *p*s < .05. Serial position effects were evident at all list lengths on which more than 1 non-focal item was presented, with the list-final item being better recalled than any of the earlier list items, all *p*s < .05. At list lengths 5 and 6, there was also a significant interaction between recall mode and serial position, due to a stronger final-item recency for immediate (probed first) than delayed (probed second) recall, *p*s < 0.05.

*Average number of items recalled at each list length*

To estimate the overall effect of list length on performance, we calculated the number of focal items recalled in the focal-only recall mode and the number of non-focal items recalled in non-focal first recall mode. A 2 x 4 repeated measures ANOVA on these two dependent variables, with the factors of item type (focal or non-focal) and list length revealed a significant main effect of item type, *F*(1, 12) = 11.457, *p* = .005, *MSE* = 0.263, with superior recall from the focal set than the non-focal set at all list lengths, and a significant main effect of list length, *F*(3, 36) = 65.858, *p* < .001, *MSE* = 0.052, with fewer correct recalls at longest lists. However, these were qualified by a significant interaction, *F*(3, 36) = 13.861, *p* < .001, *MSE* = 0.081. This reflected consistent list-length effects for focal items, all *p*s < .05, but no difference in non-focal recall between list lengths 3 and 4, *p* > .05, and a decline in non-focal recall when increasing list length from 5 and 6, *p*s < .01.

*Probability of first recall of focal and non-focal items*

Figure 2 shows the likelihood of commencing recall output with the first presented item in a given set (focal or non-focal) when this set was probed first (including focal recall data only from the focal-only mode). These data were examined across focal and non-focal items using a 2 x 4 repeated measures ANOVA with item type (focal or non-focal) and list length as factors. This revealed a significant main effect of item type, *F*(1, 12) = 9.500, *p* = .009, *MSE* = 0.092, as children were more likely to recall the first focal item first than they were to recall the first non-focal items first. There was also a significant main effect of list length, *F*(3, 36) = 66.321*, p* < .001, *MSE* = 0.031, since the probability of initially recalling the first item declined as list length increased, *p*s < 0.01. The interaction between factors was not significant, *F*(3, 36) = 2.241, *p* = 0.100, *MSE* = 0.036.

Pilot Study 2

This second experiment investigated a probed free recall (split span) task to identify a further measure of primary memory capacity in children. As with Experiment 1, the major aim was to characterize performance on this task and thus justify its use in Experiment 3 as yielding an index of primary memory. Memory items from a single list were presented as two distinct sub-sets, signified by different cartoon characters. We then probed for recall of each sub-set, varying the order of report. It was predicted that recall of the first sub-set of items (Set A) would suffer in comparison to the second sub-set of items (Set B), due to the intervening *presentation* of Set B items. In addition, we expected a recall advantage to a recall set when probed first rather than second due to *output delay / interference* (cf. Raymond, 1969).

As list lengths increase, there is a decreasing probability that primary memory capacity can represent Set B items. Thus, the point at which recall of Set B (when recalled first) is equivalent to Set A (when recalled first) should signal that primary memory capacity has been exceeded. In addition, because all items are focal items (cf. Experiment 1), recall of Set A provides one potential index of children’s secondary memory capacity, as does recall of Set B when probed second.

We also examined the effect of age on capacity. The total number of items presented to children was varied dependent on their age, as it was unknown how children aged 5-6 and 7-8 would cope with the demands of this task. The resulting predictions of this experiment were that; items in Set B when Set B was recalled first, would be recalled more accurately than items in Set A when Set A was recalled first. The degree of this differential effect would be expected to increase with age, if primary memory capacity is age dependent. Items in Set A, whether recalled first or second, would be poorly recalled, and any increase in the level of recall of Set A items with age would be indicative of the development of secondary memory capacity.

*Method*

*Participants*

Parental consent was obtained from a total of 46 pupils at a local school. Data from 23 Year 1 pupils (10 males, mean age 5 years 10 months, range 5 years 4 months to 6 years 3 months) and 23 Year 3 pupils (9 males, mean age 7 years 9 months, range 7 years 5 months to 8 years 4 months) were analyzed. All children who returned full parental consent were tested and no exclusionary criteria were applied.

*Design*

Thirty trials in total were administered. Children in Year 1 were presented with 6 item lists, which were divided into sub-sets with n items in Set A and m in Set B where n+m = 6. This allowed for five different presentation mode conditions, 5:1, 4:2, 3:3, 2:4, and 1:5 (number in first set: number in second set). Children in Year 3 were presented with 8 item lists, which were again allocated to five different presentation mode conditions, 2:6, 3:5, 4:4, 5:3, 6:2. The order of presentation of trials in each presentation mode was pseudo-randomly organized so that the number of items in each half of the list could not be anticipated. There were two conditions of recall mode, the first of which involved probing the first half items followed by the second half items, and the second of which probed the second half items followed by the first half items. Recall mode was also pseudo-randomly varied across trials, so that children did not focus preferentially on one half of the list.

*Materials and Procedure*

Children were tested in one session lasting approximately 20 minutes, and were introduced to the two cartoon characters, SpongeBob and Patrick. SpongeBob and Patrick were identified by different illustrations and two distinct male voices, and presented one sub-set of the list each (SpongeBob Set A, Patrick Set B). The task was presented on an Apple MacBook computer through internal speakers. Words for the task were chosen from a pool of 126 concrete nouns selected from a set used by Morrison, Chappell and Ellis (1997). All words selected were all acquired by 75% of children by the age of 6 years. All items were used twice and were presented both auditorily and pictorially. Both the pictorial and auditory forms of each item were presented simultaneously, at the rate of one item per second (750 ms presentation + 250 ms ISI), with no temporal distinction between sub-sets. The pictorial representations of the items consisted of black-and-white drawings, the majority of which were adapted from Snodgrass and Vanderwart (1980). An additional 57 drawings not provided by the Snodgrass set were constructed.

After the word list had been presented, children were probed to recall either SpongeBob or Patrick’s words first, and this was done by presentation of the relevant character on the screen. They were told they could recall that character’s words in any order, but should not recall the other character’s words at that time. Immediately after children felt that they had recalled all they could from the first probe, they were then prompted for the remaining character’s words with an image of that character appearing on the screen in response to a key press from the experimenter.

*Results*

Analysis was initially split by year group due to the difference in overall list length between groups. Three sets of analyses are reported; the first examining the impact of presentation and recall mode on serial position curves, the second examining the impact of presentation and recall mode on the average number of items recalled, and the third examining the probability of recalling the first item first in each set by recall mode.

*Serial position curves*

Serial position curves are reported for younger children in Figure 3a, and older children in Figure 3b. As list length varied with age, recall was analyzed separately for each age group as well as each different set length. Because the length of Set A and Set B are necessarily related (e.g., for Year 1 children, when there were 5 items in Set A there was 1 item in Set B), separate analyses were also performed for recall of each set (A or B), rather than including set as a factor in these analyses. Summary statistics for these analyses are presented in Table 4 (Table 4a for Year 1 individuals, Table 4b for Year 3 individuals).

The key findings of these analyses are that, for Set B items, there was no significant interaction between recall mode and serial position when there were 1 and 2 items in the set for Year 1 children, or when there were 2 and 3 items in the set for Year 3 children. A significant interaction between recall mode and serial position *was* observed on all longer set lengths in both age groups. This reflected marked recency effects when Set B items were recalled first, in contrast to relatively flat serial position curves when Set B items were recalled second (see Figure 3). On the lists where there was no significant interaction between recall mode and serial position, there was still a significant main effect of recall mode, with Set B items recalled first always being better recalled than those recalled second, *p*s < .05.

Turning to the analysis of Set A recall, the key points to emphasize are that among Year 1 children, there were significant serial position effects when there were 3, 4, and 5 items in a set, due to marked recency in the serial position curves (see Figure 3a). This recency effect interacted with recall mode when there were 4 or 5 items in Set A, with recency being more evident when items were recalled first at these set lengths. One possible reading of this result is that the final Set A items were held in primary memory along with the Set B items. There were significant main effects of recall mode only when there were 4 or 5 items in Set A, as items recalled first in these conditions were generally recalled better than those recalled second, but this was largely an effect of improved recall of the list final item impacting on mean recall of the list as a whole, due to the small number of items to be recalled from Set B (either 1 or 2).

In a similar vein, recall of Set A items by older children revealed significant serial position effects when there were 4 or 6 items in Set A, and these effects interacted with recall mode when there were 3, 4, or 5 items in the set. These interactions again reflected a greater advantage for Set A being probed first rather than second for list-final as opposed to list-initial items (see Figure 3b). The fact that these interactions tended to occur on the longer Set A lists, which in turn were necessarily paired with the shorter Set B lists, is again consistent with the suggestion that primary memory capacity was not completely exhausted by the requirement to maintain Set B on these trials. As a result, in these presentation mode conditions final Set A items may have been held in primary memory along with the Set B items.

*Average number of items recalled*

The second analysis examined the average total number of items recalled from a given set on any trial across year groups in each set and recall mode. As the year groups were given different total list lengths (6 in Year 1 and 8 in Year 3), to equate opportunities for recall between the groups, the average total number of items recalled for each set was taken from set lengths 2, 3, 4 and 5 only, which both year groups had in common. The average total number of items recalled from a given set was therefore calculated from 12 trials in each set and recall mode, and a 2 x 2 x 2 mixed ANOVA was conducted with year group as a between participants factor, and set (A or B), and recall mode (first or second for the set in question) as within participants factors.

This analysis revealed a non-significant main effect of year group, *F*(1, 44) = 0.341, *p* = .562, *MSE* = 0.239. The main effect of set was significant, *F*(1, 44) = 350.446, *p* < .001, *MSE* = 0.129, with more items recalled from Set B (*M* = 1.594, SD = 0.348) than Set A (*M* = 0.604, SD = 0.260). The main effect of recall mode was also significant, *F*(1, 44) = 126.519, *p* < .001, *MSE* = 0.153, with the average number of items recalled first (*M* = 1.424, SD = 0.367) being greater than the number recalled second (*M* = 0.775, SD = 0.241). There was also a significant interaction between set and recall mode, *F*(1, 44) = 96.204, *p* < .001, *MSE* = 0.087, as the advantage to recalling items first was greater in Set B than Set A (mean difference between first and second recall in Set A = 0.223, *SD* = 0.384, mean difference in Set B = 1.076, *SD* = 0.574). Crucially, the interaction between year group and set was significant, *F*(1, 44) = 5.559, *p* = .023, *MSE* = 0.129, as the difference between superior Set B recall and inferior Set A recall was more marked among Year 3 children (*M* = 1.115, *SD* = 0.409) than it was among Year 1 individuals (*M* = 0.865, *SD* = 0.300). However, Year 3 individuals did not recall significantly more items than Year 1 children on either Set A, *F*(1, 44) = 1.168, *p* = -.286, *MSE* = 0.067, or Set B, *F*(1, 44) = 2.748, *p* = .105, *MSE* = 0.116, The remaining interactions in the omnibus analysis were not significant.

In summary, more items on average were recalled from Set B than Set A, but this was particularly the case when set B was probed first. As the difference between recall modes was more marked in Set B than Set A, this suggests that Set B was more prone to output interference. This, in turn, is an indicator that Set B items were held in primary memory. However, the year groups did not differ significantly in their recall of Set B items in this analysis. This may reflect the fact that the older children received a longer total list of items, coupled with the fact that Set A and Set B items were probed equally often. As a result, older children may have been attempting to hold more items in primary memory on any given trial, a potential confound that we address in Experiment 3 below.

*Probability of recalling the first item first*

The final analysis examined the likelihood of recalling the first item in a set first at the start of recall, see Figure 4. The analysis of these data again equated set list lengths between the year groups by taking the average probability of first recall of the first item in set lengths 2, 3, 4, and 5 only, as above. A 2 x 2 x 2 mixed ANOVA was conducted on these data with year group as a between participants factor, and set (A or B), and recall mode (first or second recall of the given set) as within participants factors.

This analysis revealed a significant main effect of year group, *F*(1, 44) = 9.990, *p* = .003, *MSE* = 0.024, with children in Year 3 (*M* = 0.261, *SD* = 0.084) more likely to begin their recall from the first item in a set than children in Year 1 (*M* = 0.189, *SD* = 0.069). Overall, there was a significant main effect of set, *F*(1, 44) = 25.634, *p* < .001, *MSE* = 0.022, with children more likely to start from the start of the set in Set B (*M* = 0.281, *SD =* 0.121) than in Set A (*M* = 0.169, *SD* = 0.103). There was also a significant main effect of recall mode, *F*(1, 44) = 34.971, *p* < .001, *MSE* = 0.019, as participants were more likely to start from the start when they recalled the items first (*M* = 0.285, *SD* = 0.125) than second (*M* = 0.165, *SD* = 0.089). The interaction between year group and set was not significant, *F*(1, 44) = 1.059, *p* = .309, *MSE* = .022. The interaction between set and recall mode was significant, *F*(1, 44) = 8.313, *p* = .006, *MSE* = 0.018, but was qualified by a significant three-way interaction between all factors, *F*(1, 44) = 4.241, *p* = .045, *MSE* = 0.018. The interaction between set and recall mode was not significant among Year 1 children, *F*(1, 22) = 0.308, *p* = .584, *MSE* = .020, but was significant among Year 3 children, *F*(1, 22) = 13.592, *p* = .001, *MSE* = .016. In this older group the advantage to recalling first compared to second was smaller in Set A (mean difference = 0.033, *SD =* 0.214) than in Set B (mean difference = 0.2268 *SD* = 0.195).

In summary, older children were more likely to start recall from the start of a set than were younger children. Children were also more likely to begin their recall from the start of the set when recalling the just presented Set B items than when recalling Set A, particularly when Set B was recalled first; this effect was particularly marked among the Year 3 children. This suggests that Set B items were more likely to be maintained in primary memory than the items in Set A, particularly when Set B was probed first, leading to participants being more likely to attempt to recall Set B items in correct serial order in this instance.

Table S1

*Structure of the Interleaved Lists Task Employed in Experiment 1 (LL = Total List Length, F = Focal Item, N = Non-Focal Item)*

| Condition | Presentation order | Description |
| --- | --- | --- |
| LL3 | F-N-F | 2 focal items to recall, 1 non-focal item |
| LL4A | F-N-F-N | 2 focal items to recall, 2 non-focal items |
| LL4B | N-F-N-F | 2 focal items to recall, 2 non-focal items |
| LL5 | F-N-F-N-F | 3 focal items to recall, 2 non-focal items |
| LL6A | F-N-F-N-F-N | 3 focal items to recall, 3 non-focal items |
| LL6B | N-F-N-F-N-F | 3 focal items to recall, 3 non-focal items |

In each condition, there were 14 trials presented in one of 3 different recall modes: 8 trials requesting focal items only, 3 trials requesting focal items followed by non-focal items, 3 trials requesting non-focal items followed by focal items.

Table S2

*Analysis of Variance Statistics for Focal Item Recall in the Interleaved Lists Task Across Each List Length When a Focal Item Was Presented First in the Sequence*

| Condition |  | *F* | *df* | *p* | *MSE* |
| --- | --- | --- | --- | --- | --- |
| LL3 | Recall mode | 9.578 | 2, 24 | .001 | 0.057 |
|  | Serial position | 0.987 | 1, 12 | .340 | 0.076 |
|  | Mode x position | 1.973 | 2, 24 | .161 | 0.022 |
| LL4A | Recall mode | 8.720 | 2, 24 | .001 | 0.076 |
|  | Serial position | 0.785 | 1, 12 | .393 | 0.068 |
|  | Mode x position | 1.717 | 2, 24 | .201 | 0.047 |
| LL5 | Recall mode | 41.689 | 2, 24 | .001 | 0.035 |
|  | Serial position | 18.999 | 2, 24 | .001 | 0.076 |
|  | Mode x position | 11.985 | 4, 48 | .001 | 0.023 |
| LL6A | Recall mode | 23.076 | 2, 24 | .001 | 0.064 |
|  | Serial position | 15.335 | 2, 24 | .001 | 0.073 |
|  | Mode x position | 5.109 | 4, 48 | .002 | 0.058 |

Table S3

*Analysis of Variance Statistics for Non-Focal Item Recall in the Interleaved Lists Task Across Each List Length When a Focal Item Was Presented First in the Sequence*

| Condition |  | *t* / *F* | *df* | *p* | *MSE* |
| --- | --- | --- | --- | --- | --- |
| LL3 | Recall mode | 5.329 | 12 | <.001 | - |
| LL4A | Recall mode | 23.958 | 1, 12 | < .001 | 0.051 |
|  | Serial position | 7.287 | 1, 12 | .019 | 0.106 |
|  | Mode x position | 0.017 | 1, 12 | .899 | 0.128 |
| LL5 | Recall mode | 22.871 | 1, 12 | < .001 | 0.031 |
|  | Serial position | 7.080 | 1, 12 | .012 | 0.095 |
|  | Mode x position | 14.828 | 1, 12 | .002 | 0.027 |
| LL6A | Recall mode | 6.543 | 1, 12 | .025 | 0.063 |
|  | Serial position | 24.477 | 2, 24 | < .001 | 0.056 |
|  | Mode x position | 4.691 | 2, 24 | .019 | 0.042 |

Table S4a

*Analysis of Variance Statistics for Year 1 Individuals, Across Both Memory Sets, and Over All Set Lengths in the Split Span Task; RM = Recall mode; SP = Serial position*

* Statistics for analyses with set lengths 1 are *t*-test values.

| Set |  | Set A | | | | Set B | | | |
| --- | --- | --- | --- | --- | --- | --- | --- | --- | --- |
| length |  | *F* | *df* | *p* | *MSE* | *F* | *df* | *p* | *MSE* |
| 5 | RM | 19.432 | 1, 22 | <. 001 | 0.038 | 35.220 | 1, 22 | < .001 | 0.079 |
|  | SP | 15.016 | 4, 88 | < .001 | 0.051 | 23.437 | 4, 88 | < .001 | 0.076 |
|  | RMxSP | 5.935 | 4, 88 | < .001 | 0.049 | 5.045 | 4, 88 | .001 | 0.059 |
| 4 | RM | 5.455 | 1, 22 | .029 | 0.040 | 36.674 | 1, 22 | < .001 | 0.055 |
|  | SP | 4.035 | 3, 66 | .011 | 0.043 | 32.853 | 3, 66 | < .001 | 0.075 |
|  | RMxSP | 8.385 | 3, 66 | < .001 | 0.037 | 13.669 | 3, 66 | < .001 | 0.052 |
| 3 | RM | 1.551 | 1, 22 | .226 | 0.052 | 42.479 | 1, 22 | < .001 | 0.124 |
|  | SP | 3.406 | 2, 44 | .042 | 0.035 | 5.397 | 2, 44 | .008 | 0.088 |
|  | RMxSP | 0.128 | 2, 44 | .880 | 0.025 | 13.416 | 2, 44 | < .001 | 0.049 |
| 2 | RM | 1.131 | 1, 22 | .299 | 0.038 | 41.980 | 1, 22 | < .001 | 0.114 |
|  | SP | 1.303 | 1, 22 | .266 | 0.033 | 8.820 | 1, 22 | .007 | 0.060 |
|  | RMxSP | < 0.001 | 1, 22 | 1.00 | 0.040 | 0.042 | 1, 22 | .840 | 0.029 |
| 1* | RM | 0.940 | 22 | .357 | - | 8.654 | 22 | < .001 | - |

Table S4b

*Analysis of Variance Statistics for Year 3 Individuals, Across Both Memory Sets, and Over All Set Lengths in the Split Span Task*

| Set |  | Set A | | | | Set B | | | |
| --- | --- | --- | --- | --- | --- | --- | --- | --- | --- |
| length |  | *F* | *df* | *p* | *MSE* | *F* | *df* | *p* | *MSE* |
| 6 | RM | 3.343 | 1, 22 | .081 | 0.043 | 44.444 | 1, 22 | < .001 | 0.058 |
|  | SP | 7.880 | 5, 110 | < .001 | 0.067 | 51.013 | 5, 110 | < .001 | 0.048 |
|  | RMxSP | 4.818 | 5, 110 | .001 | 0.044 | 8.182 | 5, 110 | < .001 | 0.058 |
| 5 | RM | 1.613 | 1, 22 | .217 | 0.036 | 21.464 | 1, 22 | < .001 | 0.059 |
|  | SP | 0.611 | 4, 88 | .656 | 0.036 | 46.591 | 4, 88 | < .001 | 0.044 |
|  | RMxSP | 2.871 | 4, 88 | .028 | 0.039 | 8.768 | 4, 88 | < .001 | 0.058 |
| 4 | RM | 7.166 | 1, 22 | .014 | 0.061 | 40.480 | 1, 22 | < .001 | 0.126 |
|  | SP | 3.940 | 3, 66 | .012 | 0.043 | 12.947 | 3, 66 | < .001 | 0.063 |
|  | RMxSP | 4.046 | 3, 66 | .011 | 0.030 | 8.504 | 3, 66 | < .001 | 0.062 |
| 3 | RM | 0.227 | 1, 22 | .638 | 0.057 | 48.333 | 1, 22 | < .001 | 0.150 |
|  | SP | 2.265 | 2, 44 | .116 | 0.026 | 10.850 | 2, 44 | < .001 | 0.032 |
|  | RMxSP | 12.993 | 2, 44 | < .001 | 0.026 | 0.578 | 2, 44 | .565 | 0.060 |
| 2 | RM | 3.109 | 1, 22 | .092 | 0.087 | 79.200 | 1, 22 | < .001 | 0.093 |
|  | SP | 0.281 | 1, 22 | .601 | 0.039 | < 0.001 | 1, 22 | 1.00 | 0.023 |
|  | RMxSP | 1.147 | 1, 22 | .296 | 0.052 | 0.605 | 1, 22 | .445 | 0.032 |

*RM = Recall mode; SP = Serial position.*

Figure Captions

*Figure S1:* Serial position curves for each condition of the interleaved lists task used in Experiment 1, showing recall of both focal and non-focal items. Error bars show + / - 1 standard error of the mean, as do all figures hereafter.

*Figure S2:* The probability of starting recall with item 1 in both focal and non-focal sublists (in the most common presentation order), in the interleaved lists task used in Experiment 1.

*Figure S3a:* Serial position curves for Year 1 individuals for each condition of the split span task used in Experiment 2.

*Figure S3b:* Serial position curves for Year 3 individuals for each condition of the split span task used in Experiment 2.

*Figure S4:* Probability of starting recall with item 1 of a set in the split span task used in Experiment 2.

Figure S1


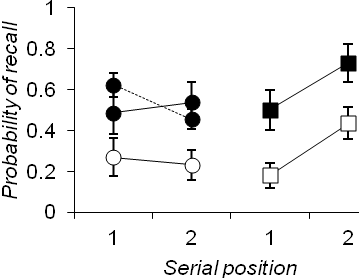

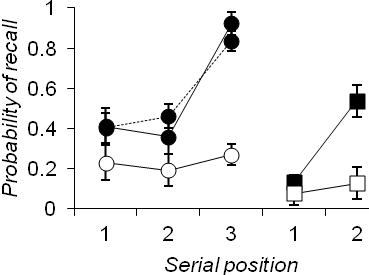

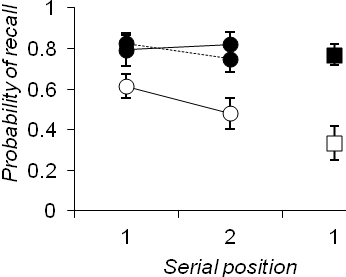

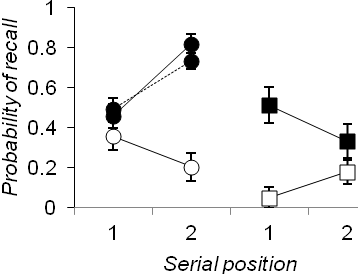


Focal alone

Focal recalled first

Focal recalled second

Non-focal recalled first

Non-focal recalled second

*Key*

*LL3A*

*LL4A*

*LL4U*

*LL5A*

*LL6A*

*LL6U*


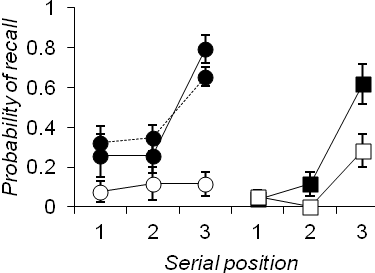

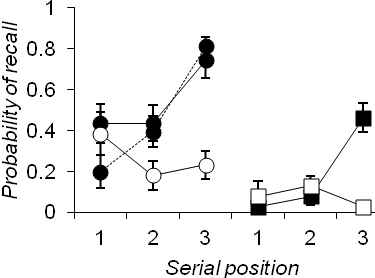


Figure S2


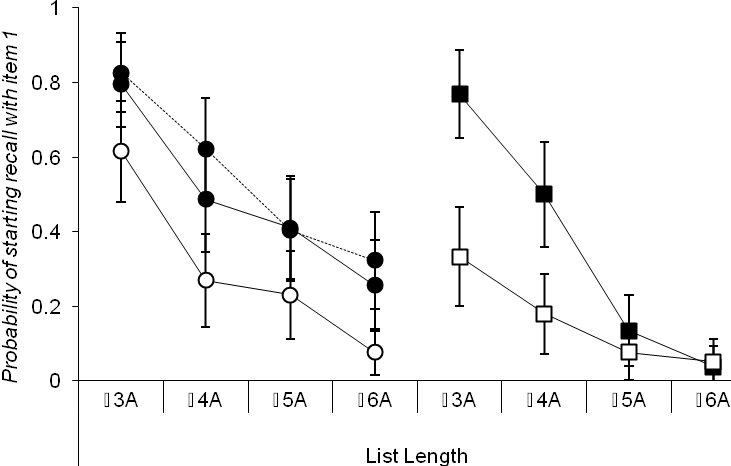


Focal alone

Focal recalled first

Focal recalled second

Non-focal recalled first

Non-focal recalled second

LL3A

LL4A

LL5A

LL6A

LL3A

LL4A

LL5A

LL6A

*Condition*

*Key*

Figure S3a


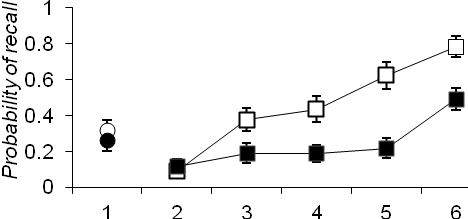


*Serial position*

1:5


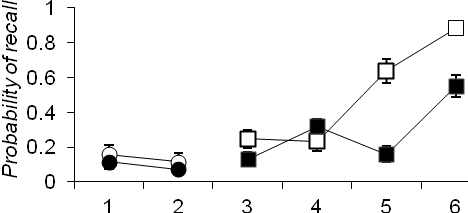


*Serial position*

2:4


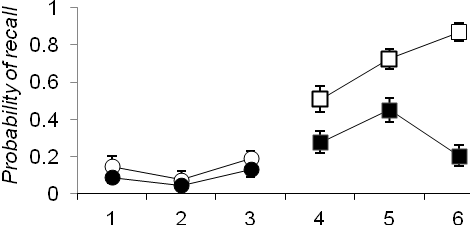


Set B recalled second

Set B recalled first

Set A recalled second

Set A recalled first

*Key*

*Serial position*

3:3


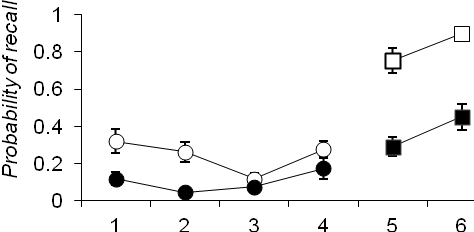


*Serial position*

4:2


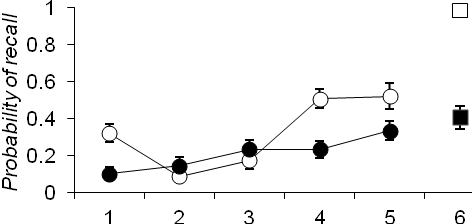


*Serial position*

5:1

Figure S3b


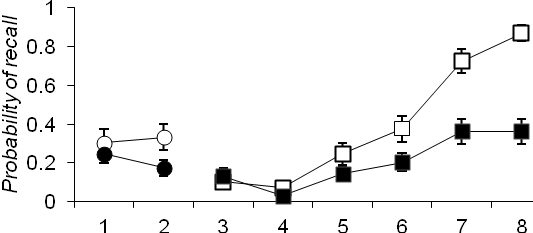


2:6

3:5


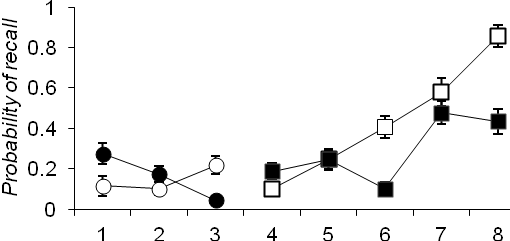


*Serial position*

*Serial position*


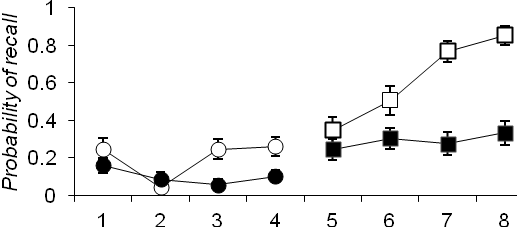


Set B recalled second

Set B recalled first

Set A recalled second

Set A recalled first

*Key*

4:4

*Serial position*


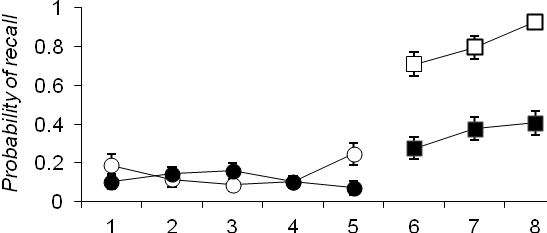


5:3

*Serial position*


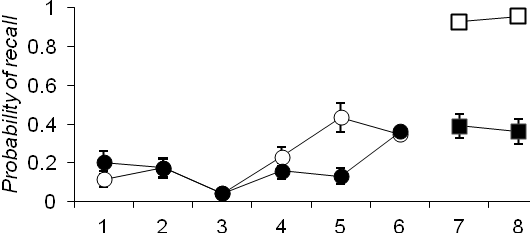


6:2

*Serial position*

Figure S4


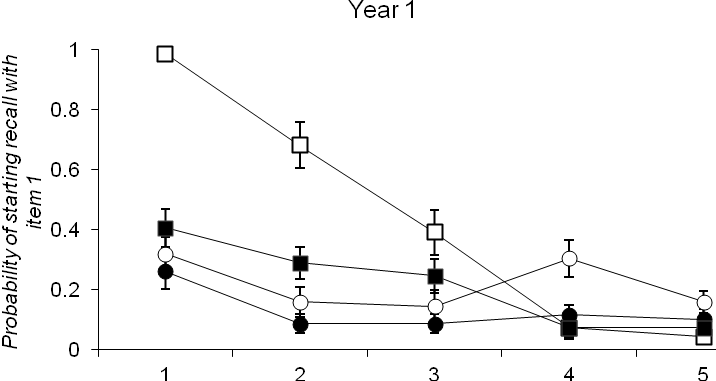


*Number of items in set*


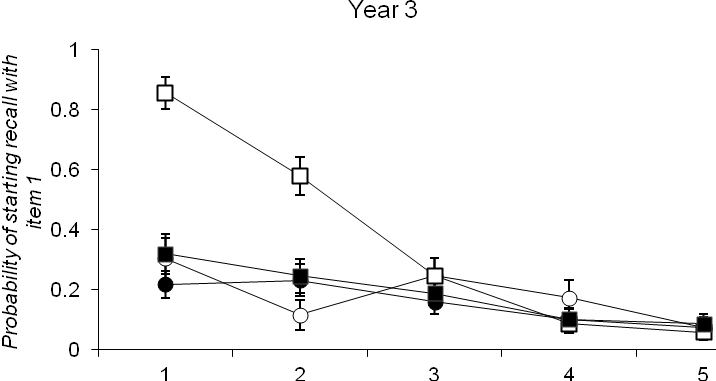


*Number of items in set*

2 3 4 5 6

Set B recalled second

Set B recalled first

Set A recalled second

Set A recalled first

*Key*
